# An immunosuppressive vascular niche drives macrophage polarization and immunotherapy resistance in glioblastoma

**DOI:** 10.1126/sciadv.adj4678

**Published:** 2024-02-28

**Authors:** Fan Yang, Md Naushad Akhtar, Duo Zhang, Rakan El-Mayta, Junyoung Shin, Jay F. Dorsey, Lin Zhang, Xiaowei Xu, Wei Guo, Stephen J. Bagley, Serge Y Fuchs, Constantinos Koumenis, Justin D. Lathia, Michael J. Mitchell, Yanqing Gong, Yi Fan

**Affiliations:** ^1^ Department of Radiation Oncology, University of Pennsylvania, Philadelphia, PA 19104, USA.; ^2^ Department of Bioengineering, University of Pennsylvania School of Engineering and Applied Science, Philadelphia, PA 19104, USA.; ^3^ Department of Obstetrics and Gynecology, University of Pennsylvania, Philadelphia, PA 19104, USA.; ^4^ Department of Pathology, University of Pennsylvania, Philadelphia, PA 19104, USA.; ^5^ Department of Biology, University of Pennsylvania, Philadelphia, PA 19104, USA.; ^6^ Abramson Cancer Center, University of Pennsylvania, Philadelphia, PA 19104, USA.; ^7^ Department of Biomedical Sciences, School of Veterinary Medicine, University of Pennsylvania, Philadelphia, PA 19104, USA.; ^8^ Lerner Research Institute, Cleveland Clinic, Cleveland, OH 44195, USA.; ^9^ Department of Medicine, University of Pennsylvania, Philadelphia, PA 19104, USA.

## Abstract

Cancer immunity is subjected to spatiotemporal regulation by leukocyte interaction with neoplastic and stromal cells, contributing to immune evasion and immunotherapy resistance. Here, we identify a distinct mesenchymal-like population of endothelial cells (ECs) that form an immunosuppressive vascular niche in glioblastoma (GBM). We reveal a spatially restricted, Twist1/SATB1-mediated sequential transcriptional activation mechanism, through which tumor ECs produce osteopontin to promote immunosuppressive macrophage (Mφ) phenotypes. Genetic or pharmacological ablation of Twist1 reverses Mφ-mediated immunosuppression and enhances T cell infiltration and activation, leading to reduced GBM growth and extended mouse survival, and sensitizing tumor to chimeric antigen receptor T immunotherapy. Thus, these findings uncover a spatially restricted mechanism controlling tumor immunity and suggest that targeting endothelial Twist1 may offer attractive opportunities for optimizing cancer immunotherapy.

## Teaser

An immunosuppressive vascular niche educates macrophage immunity and drives tumor resistance to immunotherapy.

## INTRODUCTION

Tumor immune response is a spatially regulated complex process, controlled by dynamic interaction of immune cells with neoplastic and stromal cells in the tumor microenvironment. The locoregionally dysfunctional interaction shapes the immune microenvironment, driving immune suppression and evasion in tumors and forming a major therapeutic barrier to efficient T cell–based immunotherapies. Hence, malignant solid tumors remain a substantial challenge for adoptive cellular therapy with engineered T cells, including those expressing chimeric antigen receptor (CAR), largely due to the immunosuppressive microenvironment that suppresses T cell infiltration into and activation at the tumors. Therefore, the understanding of the mechanisms controlling microenvironmental regulation of tumor immunity may help the development of therapeutic strategies to overcome tumor resistance to immunotherapy. Our recent studies show an emerging role for tumor-associated endothelial cells (ECs) in the regulation of macrophage (Mφ) function and T cell recruitment (*1*–*3*), suggesting that vascular niche may spatially orchestrate cancer immunity and render tumors resistant to immunotherapy.

Glioblastoma (GBM) is the most common and most aggressive malignant primary brain tumor in adults, with a median overall survival of about 14 to 18 months (*4*). Among the most lethal human malignancies, GBM is highly resistant to cytotoxic treatments and molecularly targeted therapies (*5*). GBM tumors are also generally refractory to T cell–based immunotherapies including PD1/PD-L1–targeting immune checkpoint blockade and CAR T cell immunotherapy (*6*–*9*), largely due to their immunologically cold nature, i.e., characterized by a paucity of tumor T cell infiltrates that result from the immunosuppressive microenvironment. These pro-tumor immune phenotypes are characterized by extraordinary abnormality of tumor vasculature and prominent infiltration with immunosuppressive Mφs, two pathognomonic and diagnostic features of GBM pathology (*5*).

Here, we sought to delineate the pivotal mechanisms underlying the generation of the immune suppressive microenvironment in GBM tumors. We report the identification of a distinct mesenchymal-like population of tumor ECs that form an immunosuppressive vascular niche for Mφ polarization through a Twist1/AT-rich sequence binding protein 1 (SATB1)/osteopontin (OPN)–dependent mechanism. Genetic or pharmacological ablation of Twist1 reverses tumor immune suppression and circumvents tumor resistance to CAR T cell immunotherapy. These findings may provide spatial insight into niche regulation of tumor immunity via EC-Mφ interaction and suggest endothelial Twist1 as a vital target for cancer immunotherapy.

## RESULTS

### A mesenchymal-like population of GBM ECs with up-regulated Twist1 expression induces alternative Mφ polarization

**Fig. 1.**
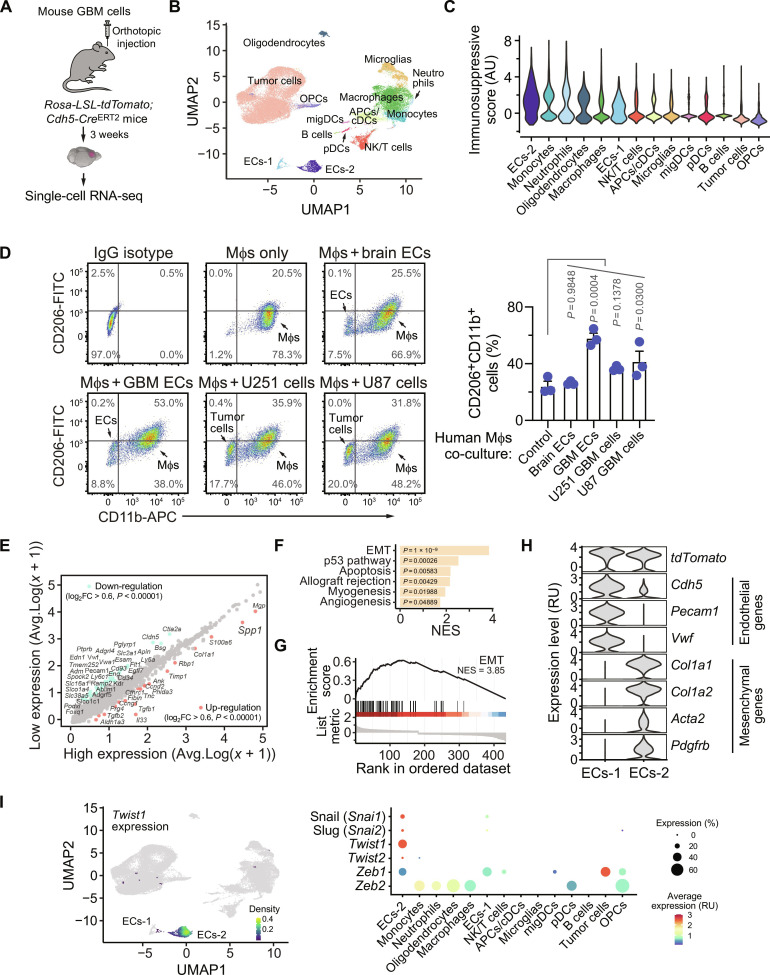
A mesenchymal-like population of ECs with Twist1 expression induces Mφ immunosuppression in GBM. (**A** to **C**) Tumor cell spheres, derived from genetically engineered mouse GBM tumors in *Ntv-a*;*Ink4a-Arf*^−/−^;*Pten*^fl/fl^;*LSL-Luc* mice, were transplanted into *Rosa-LSL-tdTomato*;*Cdh5-Cre*^ERT2^ mice. Tumors were excised and analyzed by scRNA-seq (*n* = 3 mice). (A) Experimental procedures. (B) Uniform Manifold Approximation and Projection (UMAP) analysis of transcriptome gene signature assigned cells into different clusters. (C) Immunosuppressive score in different cell clusters was analyzed on the basis of the average expression of immunosuppressive molecules. (**D**) Human peripheral blood mononuclear cell (PBMC)–derived Mφs were incubated with ECs derived from human normal brain or GBM tumors, or GBM tumor cells, followed by flow cytometry analysis. Left: Representative sortings. Right: Quantified results (*n* = 3 human samples, means ± SEM). Statistical analysis by one-way ANOVA. (**E** to **I**) GBM was induced in *Rosa-LSL-tdTomato*;*Cdh5-Cre*^ERT2^ mice, followed by scRNA-seq analysis (*n* = 3 mice). [(E) to (G)] Genes with altered expression were identified in tumor ECs with high immunosuppressive scores, compared with ECs with low immunosuppressive scores. (E) Top up-regulated and down-regulated genes. (F) Normalized enrichment scores (NES) were calculated for top enriched pathway analysis. (G) Enrichment analysis of EMT pathway. (H) Expression distribution of endothelial- and mesenchymal-associated genes in two tdTomato^+^ EC populations. (I) The expression distribution of EMT-associated transcriptional factors was analyzed in all cell clusters. Left: UMAP analysis of *Twist1* expression. Right: Expression profiles of *Snai1*, *Snai2*, *Twist1/2*, and *Zeb1/2*.

To explore expression signatures of immunosuppressive-like genes across different cell populations within GBM tumors, we performed a single-cell RNA sequencing (scRNA-seq) analysis of genetically engineered murine GBM tumors that were generated by Replication-competent avian sarcoma-leukosis virus long terminal repeat with splice acceptor (RCAS)/N-tva–mediated somatic *Pdgfb* gene transfer in *Ink4a-Arf*^−/−^;*Pten*^−/−^ neural stem/progenitor cells (Fig. 1A) (*2*, *10*, *11*). We took advantage of a genetic endothelial lineage tracing system, based on tdTomato expression driven by EC-specific *Cdh5* promoter (Fig. 1A), in which ECs are fluorescently labeled independent of EC-specific surface marker expression that may be altered by endothelial plasticity in cancer (*10*, *12*). Nonlinear dimensionality reduction by Uniform Manifold Approximation and Projection (UMAP) analysis of the whole-transcriptome gene signature assigned the single cells into several transcriptionally distinct clusters, including tumor cells, oligodendrocytes, immune cells, and two subpopulations of tdTomato^+^ ECs (ECs-1 and ECs-2) (Fig. 1B and fig. S1). We then calculated the immunosuppressive scores based on the average expression of common immunosuppressants including arginase 1 (Arg 1), Fas, FasL, interleukin-4 (IL-4)/-6/-10/-13/-33/-35/-37, transforming growth factor–β1 (TGF-β1)/-2/-3, and prostaglandin E synthase 2 in each cell population. Unexpectedly, the endothelial cluster ECs-2, followed by several myeloid cell clusters, appeared to be potentially the most immunosuppressive cell populations (Fig. 1C). These findings in mouse model faithfully recapitulated results of meta-analysis of scRNA-seq data from GBM tumors of 27 human patients; this analysis identified a similar endothelial subpopulation ECs-2 among the top in the list of cell clusters with most immunosuppressive scores (fig. S2, A and B). Considering a crucial role for tumor Mφs in GBM immunosuppression (*13*), we next tested the effects of coculturing the GBM patient tumor-derived ECs on Mφ expression of CD206, a surface marker of immunosuppressive M2 alternatively polarized Mφs. Our data showed that GBM-derived ECs robustly induced M2-like Mφ phenotypes, greater than normal brain-derived ECs or GBM tumor cells (Fig. 1D), suggesting intratumoral ECs as a putatively major cell source for Mφ polarization and GBM immunosuppression.

To characterize this immunosuppressive ECs-2 subpopulation, we analyzed top regulated genes in those tumor ECs with high or low immunosuppressive scores in the scRNA-seq data (Fig. 1E). Further gene set enrichment analysis of these altered genes revealed epithelial-mesenchymal transition (EMT) as the top regulated pathway (Fig. 1, F and G), implicating that cells in the ECs-2 cluster may undergo endothelial mesenchymal transformation (Endo-MT), i.e., partial endothelial mesenchymal transition, a process we previously characterized in GBM (*14*). In accordance with these results, cells within ECs-2 cluster expressed markedly fewer endothelial-specific genes including *Cdh5* (VE-cadherin), *Pecam1* (CD31), and *Vwf*, but more mesenchymal-like genes including *Col1a1*/*2* (collagen I-a1/1), *Acta2* (smooth muscle actin-α, α-SMA), and *Pdgfrb* [platelet-derived growth factor (PDGF) receptor β] than cells in ECs-1 cluster (Fig. 1H). A similar Endo-MT gene signature was verified in the identified EC-2 subpopulation of human GBM tumors (fig. S2, C to F). Furthermore, we analyzed gene expression signature in this ECs-2 subpopulation, with an initial focus on relevant transcriptional factors, including Snail (*Snai1*), Slug (*Snai2*), Twist1/2, and Zeb1/2, that are the master regulators of EMT in epithelium (*15*–*17*). Notably, scRNA-seq analysis of mouse GBM tumors revealed that *Twist1* was highly and exclusively expressed in the ECs-2 cluster, in contrast to a more universal pattern of *Zeb1/2* expression in different cell clusters, or a much weaker expression of *Snai1*, *Snai2*, or *Twist2* in this ECs-2 cluster (Fig. 1I).

Consistent with these findings, our meta-analysis of integrated human GBM scRNA-seq data showed that *TWIST1* was predominantly expressed in the ECs-2 subpopulation and mesenchymal-like tumor cells, while *SNAI1*, *SNAI2*, *TWIST2*, and *ZEB1/2* were more broadly expressed in different cell clusters (fig. S2, G and H). Moreover, a meta-analysis of our previously published bulk RNA-seq data with human ECs derived from GBM tumors and normal brains showed robust up-regulation of Twist1 expression in tumor ECs, compared with normal brain ECs (fig. S3, A and B). Immunoblot analysis confirmed Twist1 expression in tumor ECs (fig. S3C). Meta-analysis of a recently published scRNA-seq data with human GBM ECs (*18*) verifies *TWIST1* expression in a subpopulation of tumor ECs (fig. S4). Together, these results identify a mesenchymal-like subpopulation of tumor ECs with up-regulated Twist1 expression, which potentially drives immunosuppressive Mφ polarization.

### Genetic ablation of Twist1 inhibits Mφ immunosuppression in vitro and in vivo

**Fig. 2.**
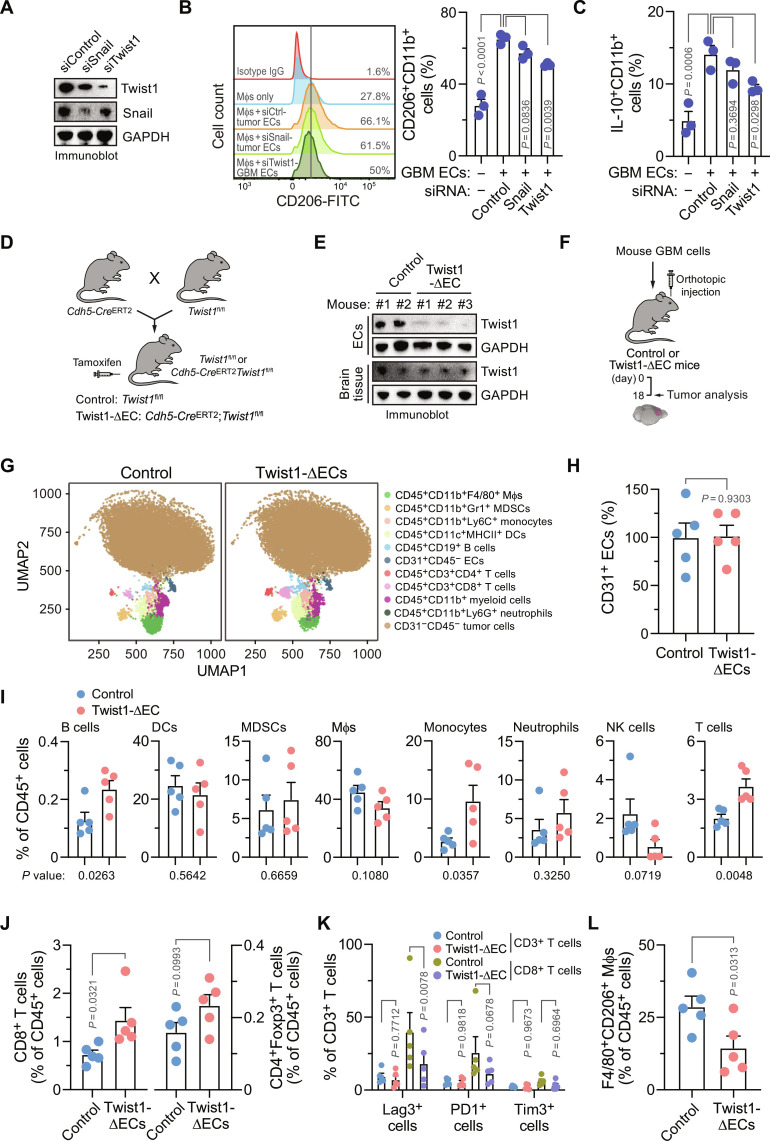
Genetic ablation of Twist1 in ECs inhibits Mφ immunosuppression in vitro and in vivo. (**A** to **C**) Human GBM ECs were treated with siRNA targeting Twist1, Snail, or control sequence. (A) EC lysates were immunoblotted. [(B) and (C)] Treated ECs were incubated with human Mφs and analyzed by flow cytometry (*n* = 3 human samples, means ± SEM). Statistical analysis by one-way ANOVA. (B) CD206^+^ cells were measured in CD11b^+^ Mφs. Left: Representative sortings. Right: Quantified results. (C) IL-10 expression in CD11b^+^ Mφs. (**D** and **E**) *Twist1*^fl/fl^ mice were crossed with *Cdh5-Cre*^ERT2^ mice to generate *Twist1*^fl/fl^ (control) or *Cdh5-Cre*^ERT2^;*Twist1*^fl/fl^ (Twist1-ΔEC) mice, followed by treatment with tamoxifen at 2 weeks old. (D) Schematic approach. (E) ECs were isolated from mouse aortas. Brain tissue and ECs were subjected to immunoblot analysis. (**F** to **L**) GBM was induced in control and Twist1-ΔEC recipient mice. Tumors were excised and analyzed by CyTOF. (F) Schematic approach. (G) representative CyTOF sortings. [(H) to (L)] Quantified CyTOF results (*n* = 5 mice, means ± SEM). Analyses for (H) ECs, (I) immune cells, (J) T effector and regulatory cells, (K) exhausted T cells, and (L) M2-polarized Mφs. [(H) to (J) and (L)] Statistical analysis by two-tailed Student’s *t* test. (K) Statistical analysis by two-way ANOVA.

We investigated the roles of endothelial Twist1 in Mφ polarization in vitro and in vivo. Our in vitro data showed that small interfering RNA (siRNA)–mediated knockdown of Twist1, but not Snail (Fig. 2A), inhibited human GBM ECs-induced immunosuppressive Mφ polarization, as indicated by reduced expressions of CD206 (Fig. 2B) and IL-10, a major immunosuppressive cytokine secreted by Mφs (Fig. 2C). These results were verified using human GBM ECs with CRISPR/single-guide RNA (sgRNA)–mediated Twist1 knockout (fig. S5).

For in vivo analysis, we crossed *Twist1*^fl/fl^ mice with *Cdh5*-*Cre* mice to generate an EC-specific Twist1 knockout mouse line, *Cdh5*-*Cre*^ERT2^;*Twist1*^fl/fl^, and used this line to investigate the role of endothelial Twist1 for tumor immunity regulation (Fig. 2D). Efficient EC-specific Twist1 knockout was verified by immunoblot analysis of isolated aortic ECs and brain tissues (Fig. 2E). GBM was then induced in these mice, followed by immunological analysis by cytometry by time of flight (CyTOF) (Fig. 2, F and G). Our data showed that genetic ablation of Twist1 in ECs did not affect the populations of ECs (Fig. 2H) but increased the infiltration of T cells into the tumors (Fig. 2I). Of note, the increase in total CD3^+^ T cells was likely attributed by cytotoxic CD8^+^ T cells rather than by CD4^+^Foxp3^+^ T_reg_ cells (Fig. 2J). Moreover, Twist1 knockout in ECs robustly reduced the expression of T cell exhaustion marker Lag3 in CD8^+^ T cells, with similar decreasing trends in PD1 and Tim3 (Fig. 2K). These results suggest that endothelial Twist inhibits T cell infiltration and induces T cell exhaustion. Consistent with our in vitro findings (Fig. 2, A to C), Twist1 deficiency in ECs reduced the cell population of F4/80^+^CD206^+^ immunosuppressive M2-like Mφs (Fig. 2L), likely contributing to the formation of a favorable microenvironment to recruit cytotoxic T cells and keep T cells less exhausted. In addition, the populations of dendritic cells (DCs), myeloid-derived suppressor cells (MDSCs), neutrophils, and natural killer (NK) cells remained unchanged in Twist knockout mice (Fig. 2I). Together, these findings suggest an important role of endothelial Twist1 for Mφ-mediated immunosuppression in GBM.

### Deletion of Twist1 in ECs inhibits tumor growth and improves animal survival

**Fig. 3.**
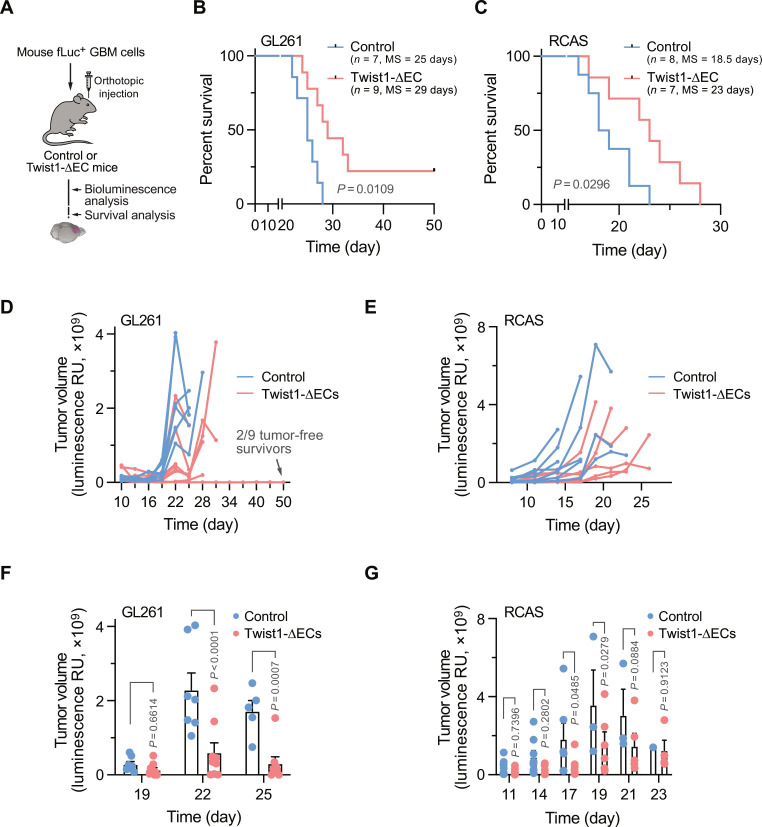
EC-specific Twist1 knockout inhibits tumor growth and improves animal survival. GBM was induced in control and Twist1-ΔEC recipient mice. (**A**) Schematic approach. (**B** and **C**) Animal survival was monitored after injection. MS, median survival. Statistical analysis by log-rank. (B) GL261 model (*n* = 7 to 9 mice). (C) RACS model (*n* = 7 to 8 mice). (**D** to **G**) Tumor growth was analyzed by whole-body bioluminescence imaging. [(D) and (E)] Tumor volume in individual mice. [(F) and (G)] Quantified results on different days (means ± SEM). Statistical analysis by two-way ANOVA. [(D) and (F)] GL261 model (*n* = 7 to 9 mice). [(E) and (G)] RACS model (*n* = 7 to 8 mice).

We next examined the effects of EC-specific knockout of Twist1 on tumor growth and animal survival in syngeneic mouse GL261 and genetically engineered RCAS models (Fig. 3A). Mice lacking Twist1 in ECs developed normally and did not exhibit any overt phenotypes. Furthermore, Twist1 deletion in ECs did not affect tumor angiogenesis or basal development, as indicated by apparently normal mouse development with unaltered vascular density manifested by numbers of CD31^+^ ECs in the tumors (Fig. 2H). Twist1 deficiency in ECs substantially improved the survival of tumor-bearing mice; more than half of Twist1 knockout mice were alive at days 28 and 23 post-tumor induction in GL261 and RCAS models, respectively, compared to the control group, where all the mice died (Fig. 3, B and C). Moreover, Twist1 knockout in ECs delayed tumor growth (Fig. 3, D and E). Notably, two of nine mice bearing GL261 tumors were tumor-free survivors when the experiments reached the endpoint at day 50, after tumors kept shrinking in the first 20 days (Fig. 3D). Similarly, average tumor sizes in control mice were two- to fourfold higher than that in Twist1-deficient mice at days 22 to 25 in the GL261 model (Fig. 3F) and at days 17, 19, and 21 in the RCAS model (Fig. 3G), collectively suggesting an important role of endothelial Twist1 in GBM growth.

### Pharmacological Twist1 inhibition reduces Mφ immunosuppression in vitro and in vivo

**Fig. 4.**
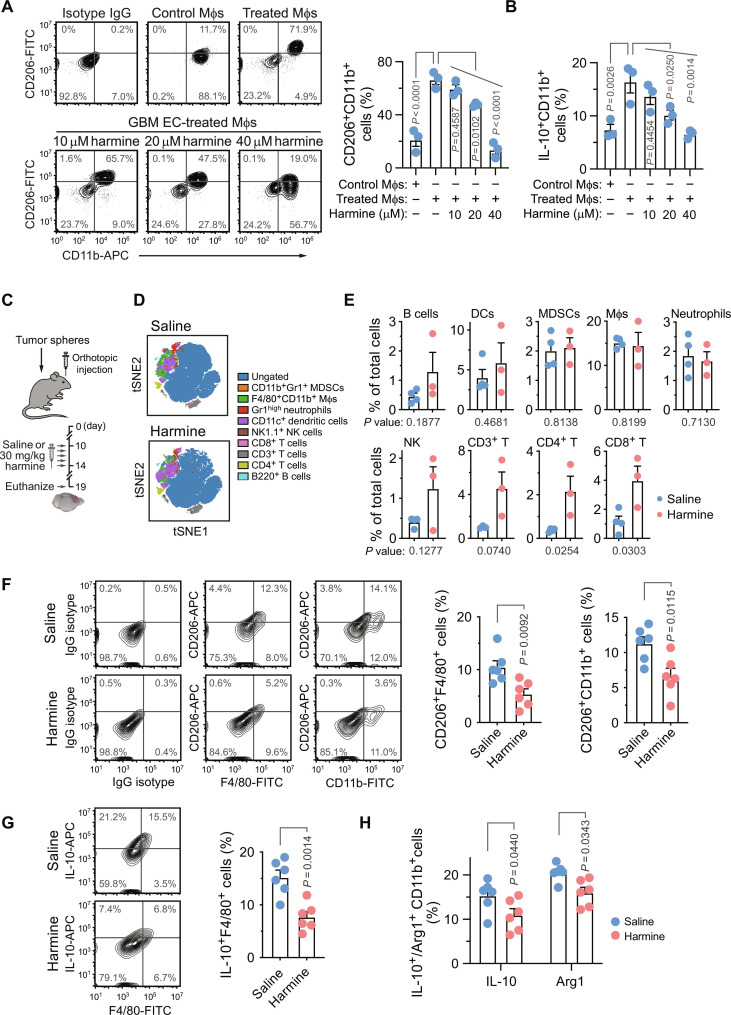
Twist1 inhibition reverses Mφ immunosuppression in vitro and in vivo. (**A** and **B**) Human PBMC-derived Mφs were incubated with or without human GBM ECs pretreated with or without harmine, and analyzed by flow cytometry for (A) CD206 and (B) IL-10 expression in CD11b^+^ Mφs. Left: Representative sortings. Right: Quantified results (*n* = 3 humans, means ± SEM). Statistical analysis by one-way ANOVA. (**C** to **H**) GBM was induced in immunocompetent mice, followed by saline or harmine treatment. (C) Experimental procedure. [(D) and (E)] Tumor-derived single-cell suspensions were analyzed by CyTOF. (D) Representative t-distributed stochastic neighbor embedding (tSNE) cell distribution. (E) Quantitative results (means ± SEM, *n* = 3 to 4 mice). Statistical analysis by two-tailed Student’s *t* test. [(F) to (H)] Tumor-derived cells were analyzed by flow cytometry analysis for (F) CD206 and [(G) and (H)] IL-10 and arginase 1 (Arg1) expression in tumor Mφs and myeloid cells. (G) Left: Representative cell sortings. Right: Quantitative results (*n* = 6 mice). Statistical analysis by [(E) to (G)] two-tailed Student’s *t* test or (H) two-way ANOVA.

To corroborate data from genetic experiments with a pharmacologic approach, we investigated the effects of a small-molecule Twist1 inhibitor harmine (*19*, *20*). Our data showed that pretreatment of human GBM ECs with harmine in vitro inhibited EC-induced M2-like Mφ phenotypes in a dose-dependent manner, as indicated by reduced CD206 and IL-10 expressions in harmine-treated Mφs (Fig. 4, A and B). In addition, harmine reduced Mφ expression of arginase 1 (*Arg1*), a critical immunosuppressant in the tumor microenvironment (fig. S6).

Moreover, immune profiling analysis of mouse GBM tumors by CyTOF showed that harmine treatment in vivo (Fig. 4C) stimulated T cell infiltration into the tumors, as indicated by robustly increased numbers of both CD4^+^ and CD8^+^ infiltrates. Harmine treatment did not substantially affect the recruitment of other immune compositions including B cells, DCs, MDSCs, Mφs, neutrophils, and NK cells (*P* > 0.1) (Fig. 4, D and E). To investigate the potential role of this immunosuppression reversal, we analyzed M2-like Mφ polarization in harmine-treated tumors by flow cytometry. Our results showed that harmine treatment reduced CD206 expression in F4/80^+^ Mφs and total CD11b^+^ myeloid cells in tumors (Fig. 4F). Likewise, harmine treatment suppressed the production of IL-10 and the expression of Arg1 in GBM-associated Mφs and total myeloid cells (Fig. 4, G and H). These findings indicate that pharmacological Twist1 inhibition reverses Mφ-mediated immunosuppression in GBM.

### Twist1 inhibition sensitizes GBM to CAR T cell immunotherapy

**Fig. 5.**
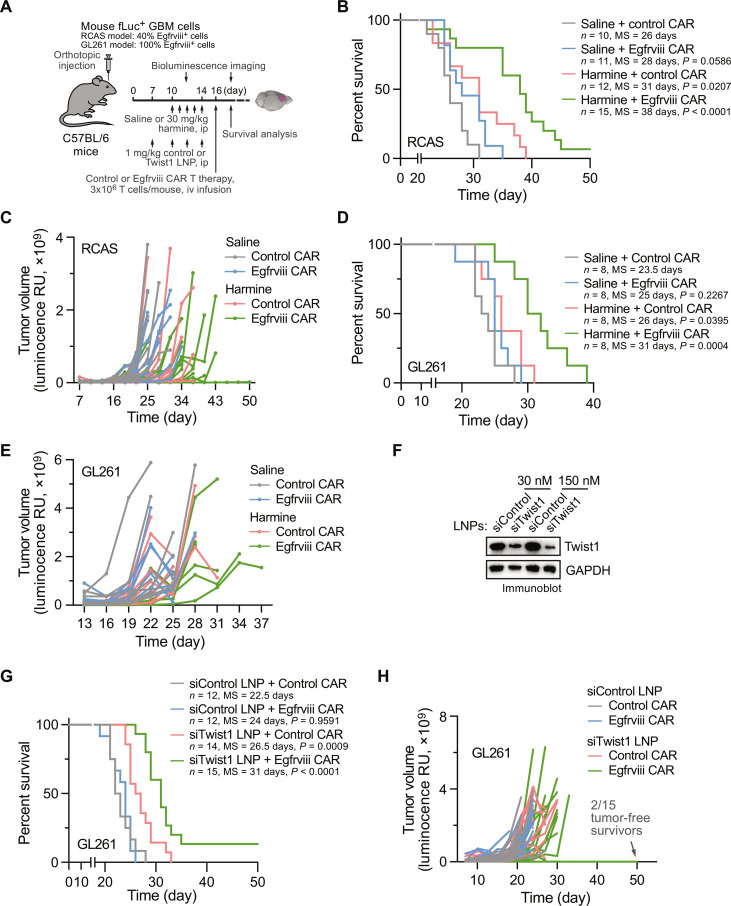
Twist1 inhibition sensitizes GBM to CAR T immunotherapy. GBM was induced in immunocompetent mice by transplantation of Egfrviii^+^;fLuc^+^ RCAS or GL261 tumor cells, followed by treatment with saline, harmine, or siRNA LNP, plus treatment adoptive CAR T cell transfer. (**A**) Experimental procedure. (**B** and **C**) RCAS model with harmine treatment (*n* = 10 to 15 mice). (B) Animal survival was monitored and analyzed by log-rank test. (C) Tumor volumes were analyzed by bioluminescence imaging. (**D** and **E**) GL261 model with harmine treatment (*n* = 8 mice). (D) Animal survival was monitored and analyzed by log-rank test. (E) Tumor volumes were analyzed by bioluminescence imaging. (**F**) Knockdown efficiency of siRNA LNP. Mouse ECs were treated with LNPs containing control or Twist1 siRNA, and cell lysates were immunoblotted. (**G** and **H**) GL261 model with siRNA LNP treatment (*n* = 12 to 15 mice) (harmine). Animal survival was monitored and analyzed by log-rank test. (H) Tumor volumes were analyzed by bioluminescence imaging.

Given enhanced T cell infiltration and reversed immunosuppression by genetic and pharmacological Twist1 ablation (Figs. 2 and 4), we hypothesize that therapeutic Twist1 inactivation may improve adoptive T cell immunotherapy. To test this hypothesis, we performed experimental therapy combining Twist1 inhibition with CAR T cell infusion, in which a fully murine system we recently developed was used to specifically target tumor cells expressing mouse Egfrviii, a hallmark of GBM-associated mutation (Fig. 5A). Our data showed that CAR T cell immunotherapy alone did not affect animal survival (*P* > 0.05) or tumor growth in the RCAS-mediated genetically engineered mouse model (Fig. 5, B and C). Consistent with the results of Twist1 knockout in ECs (Fig. 3), monotherapy with the Twist1 inhibitor harmine moderately extended mouse survival (+5 days, from control basal median survival of 26 days, *P* < 0.05). Notably, combination treatment substantially extended animal survival (+12 days, *P* < 0.001) with delayed tumor growth (Fig. 5, B and C). Of note, one-third of the mice that received combination therapy remained alive, while all mice in other groups died 39 days after tumor induction (Fig. 5B). In addition, one mouse in the combination treatment group showed a complete response, surviving to day 50 when the experiments reached the endpoint. In a parallel study, similar therapeutic results were observed in a GL261 syngeneic mouse GBM model (Fig. 5, D and E), collectively suggesting that pharmacological inhibition of Twist1 by harmine overcomes tumor resistance to CAR T cell therapy in GBM.

In addition, to exclude the possibility of therapeutic effects due to potential nonspecific or off-target activities of pharmacologic inhibition by harmine, we tested an experimental therapy by targeted delivery of Twist1 siRNA into ECs using a polymer-lipid nanoparticle (LNP)–based system we recently developed (Fig. 5, F to H) (*21*–*23*). Treatment of GBM-bearing mice with Twist1 siRNA LNP alone slightly improved animal survival (+4 days, from control basal median survival of 22.5 days, *P* < 0.001). Notably, combination treatment with Twist1 siRNA LNP and Egfrviii CAR T cells substantially extended animal survival (+8.5 days, *P* < 0.0001; Fig. 5G) and delayed tumor growth (Fig. 5H, left), showing comparable therapeutic efficacy to combination therapy with harmine and CAR T cells. Notably, after combination treatment, 2 of 15 mice were tumor-free survivors when the experiments reached the endpoint at day 50 (Fig. 5H, right). Together, these results suggest that Twist1 inhibition sensitizes GBM tumors to CAR T cell immunotherapy.

### Endothelial Twist1 promotes alternative Mφ polarization via OPN

**Fig. 6.**
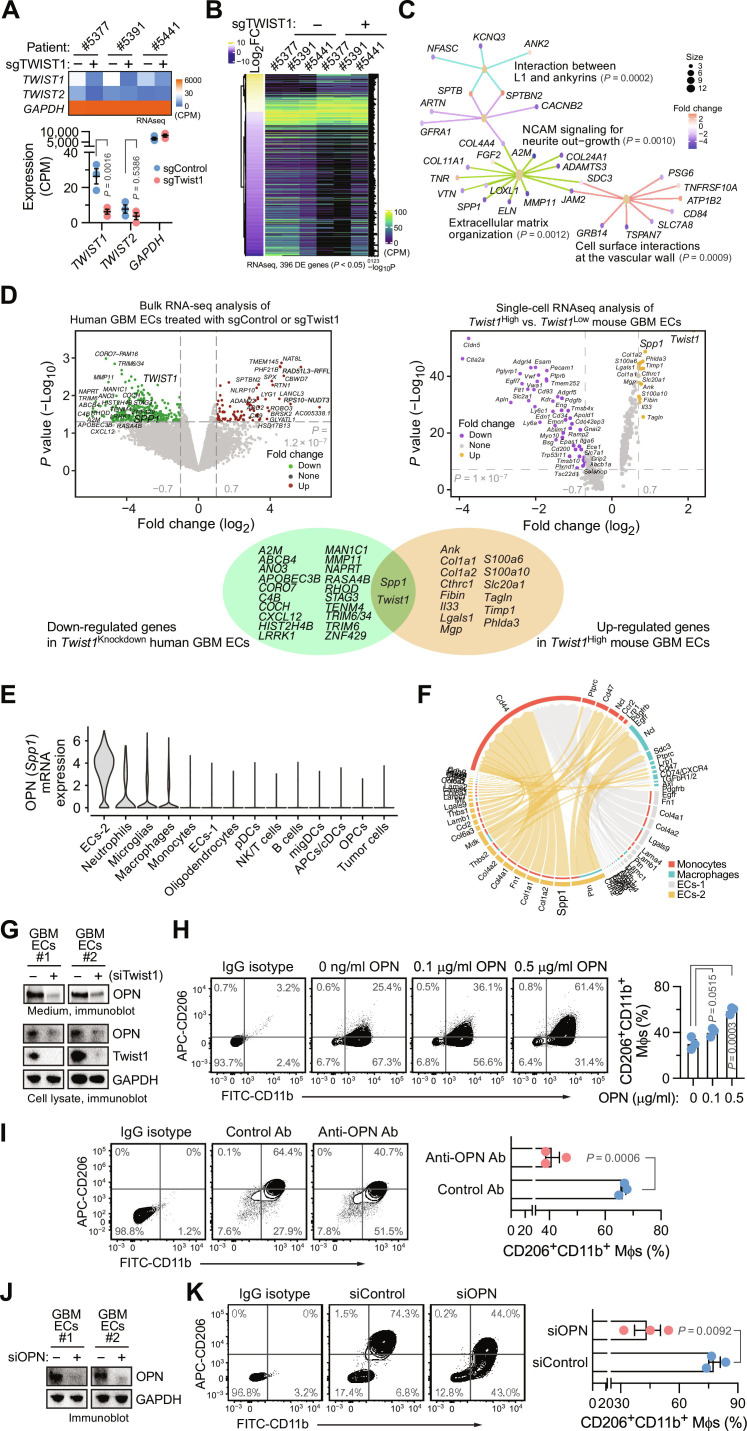
Endothelial twist1 promotes alternative Mφ polarization via OPN. (**A** to **D**) GBM ECs isolated from patients (*n* = 3) were transduced with lentivirus encoding control or Twist1 CRISPR/sgRNA, followed by RNA-seq analysis. (A) Analysis of Twist1/2 expression. Top: Heatmap of gene expression. Bottom: Quantified results (means ± SEM). Statistical analysis by two-way ANOVA. (B) Analysis of top regulated genes with differentiated expression (DE; fold change > 0.5) and *P* < 0.05. (C) Reactome analysis of top regulated genes. (D) to (**F**) Genetically engineered mouse GBM tumors were analyzed by scRNA-seq (*n* = 3). (D) Overlap in top regulated genes identified in bulk RNA-seq analysis of human GBM ECs treated with control or Twist1 sgRNA (left) and scRNA-seq analysis of mouse GBM ECs with high or low expression of *Twist1* (right). [(E) and (F)] Analysis of mouse scRNA-seq data. (E) OPN (*Spp1*) expression in different cell clusters was analyzed. (F) Potential interaction pathways between ECs and monocytes/Mφs were predicted by ligand-receptor analysis. (**G**) Human GBM ECs were treated with control or Twist1 siRNA. Cell lysates and medium were immunoblotted. (**H**) Human PBMC-derived Mφs were treated with OPN at different doses, followed by flow cytometry analysis. Left: Representative cell sortings. Right: Quantified results (means ± SEM, *n* = 3). Statistical analysis by one-way ANOVA. (**I**) Human Mφs were treated with GBM EC–derived conditioned medium in the presence or absence of anti-OPN neutralizing antibody, and analyzed by flow cytometry. Left: Representative cell sortings. Right: Quantified results (means ± SEM, *n* = 3). Statistical analysis by Student’s *t* test. (**J** and **K**) GBM ECs were treated with control or OPN siRNA. (J) Cell lysates were immunoblotted. (K) Treated ECs were incubated with human Mφs and analyzed by flow cytometry. Left: Representative cell sortings. Right: Quantified results (means ± SEM, *n* = 3). Statistical analysis by Student’s *t* test.

To gain molecular insights into the regulation of Mφ immunosuppression by endothelial Twist1, we determined transcriptome changes in tumor ECs treated with CRISPR/sgRNA targeting Twist1. Bulk RNA-seq analysis of tumor ECs derived from three human patients with GBM confirmed specific knockdown of Twist1, but not Twist2 (Fig. 6A), and identified about 400 differentially expressed genes in Twist1-knockdown ECs (Fig. 6B). Further gene set enrichment analysis of these genes revealed several pathways, including matrix organization, L1-ankyrins interaction, neural cell adhesion molecule signaling, and cell surface interaction, among the top of regulatory modulators (Fig. 6C). To identify the downstream targets regulated by Twist1, we comprehensively analyzed the top down-regulated genes in Twist1-knockdown human GBM ECs and up-regulated genes in mouse GBM ECs with high Twist1 expression. Notably, the single-gene, secreted phosphoprotein 1 (*SPP1*) that encodes cytokine osteopontin (OPN), was presented in both identified gene groups (Fig. 6D). *SPP1* was identified in the top regulated pathway “matrix organization” (Fig. 6C) and was also among the top two up-regulated genes in tumor ECs with higher immunosuppressive scores revealed by scRNA-seq analysis of mouse GBM tumors (Fig. 1E). Moreover, analysis of mouse GBM tumors showed that *Spp1 *is robustly up-regulated in the cell cluster of ECs-2 (Fig. 6E), which was characterized as a mesenchymal-like population of ECs with up-regulated *Twist1* expression and potential ability to induce Mφ immunosuppression (Fig. 1), suggesting ECs as another major source of OPN production, in addition to macrophages and glioma cells (*24*, *25*). Likewise, computational network analysis of mouse scRNA-seq data suggests *Spp1*/*Cd44* as one pair of top predicted genes of ligand-receptor interaction in tumor ECs-2 and Mφs, respectively (Fig. 6F). Furthermore, immunoblot analysis showed that siRNA-mediated knockdown of Twist1 reduced OPN expression in human GBM ECs (Fig. 6G), collectively suggesting OPN as a Twist1-regulated cytokine produced by the intratumoral ECs.

We next investigated the role of OPN in EC-mediated Mφ polarization. Our data showed that treatment with purified OPN induced immunosuppressive Mφ polarization, as indicated by a dose-dependent increase in CD206 expression in human Mφs (Fig. 6H). Antibody-based neutralization of OPN reduced GBM EC conditioned medium-induced CD206 expression in Mφs (Fig. 6I), suggesting a requisite role of OPN for EC-mediated immunosuppressive Mφ polarization. In accordance with these findings, siRNA-mediated knockdown of OPN in ECs inhibited EC-induced CD206 expression in Mφs (Fig. 6, J and K). Furthermore, neutralization of OPN rescued T cell proliferation and expression of interferon-γ (IFN-γ) in the presence of tumor EC-educated, immunosuppressive Mφs (fig. S7). Together, these results identify that OPN acts as a Twist1 downstream to mediate EC-induced pro-tumorigenic Mφ polarization.

### Twist1 and SATB1 regulate OPN expression in tumor ECs

**Fig. 7.**
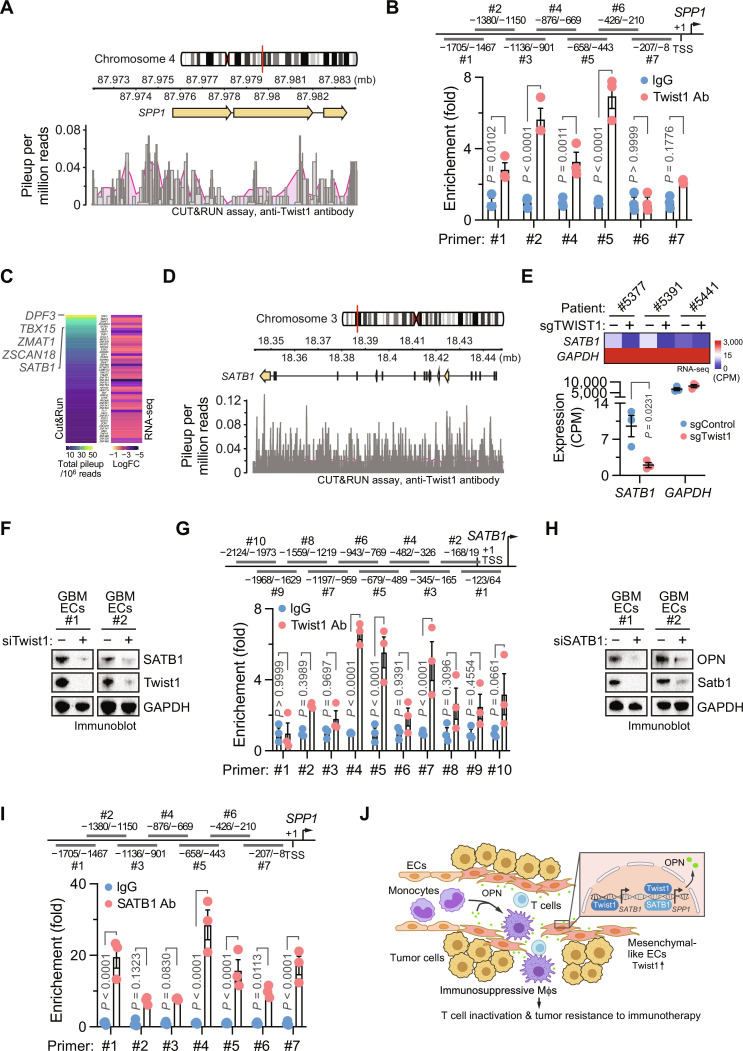
Twist1-dependent SATB1 expression induces OPN transcription in GBM ECs. (**A**) Human GBM ECs were subjected to CUT&RUN analysis with anti-Twist1 and control immunoglobulin G (IgG) antibodies. Pileup signals for Twist1 binding to the *SPP1* gene region were calculated with IgG normalization (pooled from three EC samples). (**B**) ECs were subjected to chromatin immunoprecipitation (ChIP) analysis with anti-Twist1 or IgG antibody. Top: Different primers targeting the *SPP1* gene were used. Bottom: Quantified results (means ± SEM, *n* = 3). Statistical analysis by two-way ANOVA. (**C** and **D**) ECs were subjected to CUT&RUN analysis with anti-Twist1 and control IgG antibodies. (C) ECs were transduced with lentivirus encoding control or Twist1 CRISPR/sgRNA and subjected to RNA-seq analysis. The top 50 down-regulated transcriptional factors were shown with CUT&RUN pileup signals (*n* = 3). (D) Pileup signals for Twist1 binding to the *SATB1* gene region were calculated with IgG normalization (pooled from three EC samples). (**E**) ECs were transduced with lentivirus encoding control or Twist1 CRISPR/sgRNA and subjected to RNA-seq analysis for *SATB1* expression. Top: Heatmap of gene expression. Bottom: Quantified results (means ± SEM, *n* = 3). Statistical analysis by Student’s *t* test. (**F**) ECs were treated with control or Twist1 siRNA. Cell lysates were immunoblotted. (**G**) ECs were subjected to ChIP analysis with anti-Twist1 or IgG antibodies and analyzed by ChIP analysis. Top: Different primers targeting the *SATB1* gene were used. Bottom: Quantified results (means ± SEM, *n* = 3). Statistical analysis by two-way ANOVA. (**H**) ECs were treated with control or SATB1 siRNA. Cell lysates were immunoblotted. (**I**) ECs were subjected to ChIP analysis with anti-SATB1 or IgG antibodies and analyzed by ChIP analysis. Top: Different primers targeting the *SPP1* gene were used. Bottom: Quantified results (means ± SEM, *n* = 3). Statistical analysis by two-way ANOVA. (**J**) A schematic model. Mesenchymal-like ECs form an immunosuppressive niche, inducing Mφ polarization via Twist1/SATB1-mediated OPN expression.

To explore the mechanism by which Twist1 induces OPN expression in GBM ECs, we mapped global genome binding sites of Twist1 using an approach that combines cleavage under targets and release using nuclease (CUT&RUN) with massively parallel DNA sequencing. Our data identified a binding site located at the promoter region of *SPP1* for Twist1 (Fig. 7A), suggesting that OPN transcription could be directly induced by Twist1 binding to its promoter. Consistently, our chromatin immunoprecipitation (ChIP) analysis verified the interaction between Twist1 and *SPP1* promoter DNA (Fig. 7B). To explore additional mechanisms underlying Twist1-mediated OPN expression, we further analyzed CUT&RUN data with the top 50 transcriptional factors whose expression was positively regulated by Twist1 as revealed by our bulk RNA-seq analysis of Twist1 CRISPR/sgRNA–treated human GBM ECs; our analysis identified several transcriptional factors including Dpf3, Tbx15, Zmat1, Zscan18, and SATB1 that may most robustly interact with Twist1 (Fig. 7C). Of these five transcriptional factors, SATB1 was predicted to tentatively bind to the *SPP1* promoter by an in silico binding motif analysis, implicating SATB1 as a tentative downstream transcriptional factor that may additionally contribute to Twist1-induced OPN expression. In accordance with this hypothesis, CUT&RUN analysis showed that there was a marked binding activity of *SATB1 *promoter region to Twist1 (Fig. 7D); SATB1 mRNA expression was substantially reduced by Twist1 knockdown (Fig. 7E), verified by immunoblot analysis showing that siRNA-mediated knockdown of Twist1 abrogated SATB1 expression in GBM ECs (Fig. 7F). This suggests that Twist1 may transcriptionally regulate the expression of SATB1. Furthermore, ChIP analysis showed that Twist1 interacted with *SATB1* promoter DNA in GBM ECs (Fig. 7G). siRNA-mediated knockdown of SATB1 reduced OPN expression in GBM ECs (Fig. 7H), suggesting a critical role of SATB1 in OPN expression. Likewise, ChIP analysis showed that SATB1 bound to *SPP1* promoter DNA in GBM ECs (Fig. 7I), collectively showing a transcriptional Twist1/ SATB1 axis for OPN expression. Together, these findings suggest a sequential transcriptional activation mechanism for OPN expression, driven directly by Twist1 and indirectly by Twist1-downstream SATB1 in tumor ECs (Fig. 7J).

## DISCUSSION

Immunotherapy holds great promise for treating cancer. Despite the adoptive transfer of T cells having yielded unprecedented success in a subset of human cancers, such as leukemia, immunologically cold solid tumors including GBM are largely refractory to CAR T cell therapy. This is largely due to limited T cell infiltration and activation resulting from hostile immunity in the tumor microenvironment, in which dysfunctional interaction of immune cells with neoplastic and stromal cells induces tumor immunosuppression and resistance to immunotherapy. Here, our study identifies a mesenchymal-like subpopulation of ECs as a major source of tumor immunosuppression, interacting with tumor Mφs to drive immunosuppressive phenotypes via Twist1/SATB1-dependent expression and secretion of OPN (Fig. 7J). Given that vasculature is the avenue through which circulation-derived immune cells infiltrate tumors, the vascular niche may act as an immune-education facility to modulate leukocyte functions with locally enriched EC-secreted molecules, i.e., angiocrine, in addition to its conventional role of delivery of immune cells into the tumors. Our findings illustrate a spatially regulated mechanism by which the vascular niche fuels pro-tumor immunity and suggest that targeting endothelial Twist1 may represent a promising strategy for enhancing the efficacy of T cell–based immunotherapy.

Mesenchymal-like transformation in neoplastic cells has been well characterized, contributing to tumor progression, metastasis, and resistance to cytotoxic therapy. Our study shows that a subpopulation of tumor ECs undergo mesenchymal-like transcriptional reprogramming, forming a unique immunosuppressive vascular niche to render tumors resistant to immunotherapy. ECs exhibit dynamic cell plasticity in embryogenesis and pathological settings including cardiac, renal and liver fibrosis, pulmonary hypertension, vascular inflammation, and cerebral cavernous malformation (*26*–*37*). Our recent work shows that tumor ECs undergo a mesenchymal transformation, i.e., Endo-MT, to induce Snail/Slug-dependent aberrant vascularization via HGF/c-Met–, PDGF/PDGFR-β–, and PAK4-mediated mechanisms (*2*, *10*, *14*, *38*). Our present work reveals an additional role for EC plasticity in tumor immunity regulation, acting via a Twist1-mediated paracrine effect on tumor Mφs. Twist1 knockout did not affect tumor EC proliferation, implicating a main regulatory function of Twist1 in the expression of immunosuppressive factors but not in vascular structure. Supportive to this concept of a vascular niche for Mφs, our previous work shows that tumor ECs are proximately localized near immunosuppressive Mφs, which stimulates HIF-2α/PPAR-γ–dependent Mφ alternative activation (*1*).

Tumor Mϕs exhibit immunosuppressive phenotypes, blocking anti-tumor immunity of cytotoxic T and NK cells through stimulus-dependent functional polarization (*39*–*45*). Notably, GBM is featured by extensive infiltration with myeloid cells, such as Mφs that make up as much as half of the nonneoplastic cells (*13*). In contrast to classically activated Mφs that stimulate phagocytosis, inflammation, and host immunity, a prominent population of tumor Mφs undergo alternative, M2-like polarization to express anti-inflammatory molecules, such as IL-10, TGF-β, and arginase 1 to induce tumor immunosuppression (*46*–*49*). Treatments manipulating Mφ proliferation, differentiation, and polarization pathways that are mediated through CSF-1, PI3K, TLR4, CD40, and CD47 have recently been exploited, showing limited benefits or are still under evaluation (*39*–*42*), and their therapeutic efficacy is challenged by redundancy and dispensability of these downstream pathways. Our present work reveals a mechanism that acts as an upstream determinant of functional Mφ regulation in the tumor microenvironment, namely, Twist1 in a vascular niche, serving as an alternative intervention target. We show that genetic ablation of endothelial Twist1, as well as pharmacological inhibition of Twist1, reduces Mφ-mediated tumor immunosuppression and circumvents tumor resistance to CAR T cell immunotherapy.

Twist1 is a master transcriptional factor of EMT, acting as a transcriptional repressor or enhancer (*16*, *17*, *50*, *51*). A central role Twist1 plays is to suppress the expression of E-cadherin and to promote the expression of mesenchymal-associated proteins including PDGFR-β, MMP-1, and BMI1 in tumor cells, leading to tumor progression and metastasis (*52*–*55*). Our data unexpectedly show that Twist1 is almost exclusively expressed by the immunosuppressive, mesenchymal-like subpopulation of ECs in mouse GBM tumors (and, to a lesser extent, EMT-acquiring tumor cells in human GBM tumors), suggesting Twist1 as a potential regulator of Endo-MT and vascular niche function. In accordance with the role of Twist1 in Endo-MT during cancer progression, Twist1 regulates mesenchymal phenotypes including the enhanced proliferation and migration in ECs during development and cardiovascular diseases (*56*–*58*). Furthermore, we determine that endothelial Twist1 is a requisite for immunosuppressive Mφ phenotypes, at least partially, through Twist1-transcripted OPN. Twist1 is also known to transcript CCL2 and CXCL12 that can recruit pro-tumor myeloid cells (*59*, *60*), serving as a potentially additional mechanism to induce vascular niche-mediated Mφ immunosuppression.

OPN, a secreted glycoprotein known to be expressed by epithelial and mesenchymal cells, Mφs, and neutrophils, is critical for wound healing, bone homeostasis, and cancer development and metastasis (*61*, *62*). OPN is frequently overexpressed in various cancer types (*61*, *63*), and its expression is associated with poor survival in patients with GBM (*64*). Previous studies suggest that tumor macrophages and glioma cells are major sources of OPN in GBM (*24*, *25*). Here, we show that tumor ECs most robustly express OPN in the tumor microenvironment. Considering the relatively low cell numbers of ECs, consisting of typically 5 to 10% of total nonneoplastic cells, ECs may not be the main source for OPN, but infiltrating monocytes/macrophages are educated by locally enriched OPN in the vascular niche, serving as a major driving force for M2-like Mφ polarization and GBM immunosuppression. Consistent with our findings, recent studies show that OPN stimulates pro-tumor Mφ functions through integrins α9β1 and αvβ5 (*25*, *65*). In addition, OPN directly suppresses the anti-tumor functions of CD8^+^ T cells by binding to its receptor CD44 in T cells (*66*), suggesting OPN as a multifunctional immunosuppressant in the tumor microenvironment. The molecular mechanism underlying OPN overexpression in cancer remains largely unclear, and our study uncovers a sequential Twist1/SATB1 transcriptional activation mechanism that leads to OPN expression in tumor ECs. OPN activates Akt and extracellular signal–regulated kinase to induce Twist1 expression by engaging CD44 and αvβ3 integrin (*67*), indicative of a potential positive feedback mechanism for up-regulation of OPN and Twist1.

Growing evidence suggests that SATB1 reprograms chromatin structure and transcription profile to promote tumor cell proliferation and EMT, leading to cancer progression (*68*–*71*). In addition to its oncogenic functions, SATB1 regulates cytokine expression and modulates the activity of T cells and DCs in development and cancer (*72*–*78*). Our findings, supported by these published results, suggest that SATB1 is a potential positive regulator of pro-tumor immunity driven by EC-Mφ interaction since SATB1 is required for the expression of OPN that can induce immunosuppressive Mφ activation. Furthermore, our study reveals a sequential transcription activation mechanism that leads to Twist1-inducible SATB1 transcription and SATB1-inducible OPN transcription, providing molecular insight into SATB1-mediated tumor immunity.

In sum, our work suggests a multidimensional mechanism controlling tumor immunity through spatial interaction between tumor ECs and Mφs, in which a Twist1/SATB1/OPN axis serves as a regulatory node for tumor immunosuppression. These findings highlight a distinct EC subpopulation driving tumor resistance to immunotherapy and may offer a promising strategy for therapeutic manipulation of the immunosuppressive vascular niche to fuel T cell-based immunotherapy in solid tumors.

## MATERIALS AND METHODS

### Human monocyte isolation and treatment

Peripheral blood mononuclear cell–derived monocytes were obtained from healthy human volunteer donors ages 16 to 64 of all genders, races, and ethnicities at Human Immunology Core at University of Pennsylvania. Informed consent was obtained from all donors under an Institutional Review Board–approved protocol at University of Pennsylvania. Our work with human participants complies with all relevant ethical regulations. Human primary monocytes were cultured in RPMI 1640 medium supplemented with 10% fetal bovine serum (FBS) and treated with human CSF-1 (10 ng/ml; BioLegend, 574806) for 5 days to differentiate into macrophages. Cells were treated with recombinant human OPN (R&D Systems, 1433-OP-050/CF), or cocultured (20:1) with ECs isolated from human GBM tumors, human brain microvascular ECs (ScienCell, 1000), or human glioma cells (U251 cells, Sigma-Aldrich, 09063001; U87 cells, Sigma-Aldrich, 89081402) for 2 days in the presence or absence of or OPN-neutralizing antibody (1 μg/ml; R&D Systems, AF1433-SP).

### Human GBM EC isolation and culture

Surgical specimens from human patients with GBM ages 48 to 83 in all genders, multiple races, and ethnicities were collected at the Department of Neurosurgery of the Hospital of the University of Pennsylvania. The collection of human tissues in compliance with the tissue banking protocol was approved by the University of Pennsylvania Institutional Review Board, and written informed consent was obtained from each participant. ECs were isolated and verified as previously described (*10*, *38*). Tumor-derived single-cell suspensions were prepared by the tissue bank. Cell suspensions were subjected to magnetic-activated cell sorting (MACS) with anti-CD31 antibody-conjugated magnetic beads (Miltenyi Biotech, 130-091-935). All cells were used between passages 2 and 6. ECs were treated with harmine (10 to 40 μM; Sigma-Aldrich, 286044).

### Mice

Wild-type (WT) mice on the C57BL/6J background were purchased from Jackson Lab. *Cdh5-Cre*^ERT2^;*Rosa-LSL-tdTomato* mice were generated by crossing *Rosa-LSL-tdTomato* mice (Jackson Laboratory) with *Cdh5-Cre*^ERT2^ mice (provided by R. Adams at Max Planck). *Cdh5-Cre*^ERT2^;*Twist1*^fl/fl^ mice were generated by crossing *Twist1*^flox/flox^ mice (Mutant Mouse Resource and Research Centers, RRID:MMRRC_016842-UNC) [PMID: 17868088] with *Cdh5-Cre*^ERT2^ mice. Mice (half male and half female, 2 weeks old) were intraperitoneally injected with tamoxifen (80 mg/kg) daily for 5 consecutive days. All animals were housed at room temperature with a 12/12-hour light/dark cycle in the Association for the Assessment and Accreditation of Laboratory Animal Care–accredited animal facility of University of Pennsylvania. All animal studies were reviewed and approved by the Institutional Animal Care and Use Committee at University of Pennsylvania. We have complied with all relevant ethical regulations for animal testing and research.

### GBM tumor model and treatment

A genetically engineered mouse GBM model was induced as previously described (*10*, *79*). Briefly, chicken DF-1 fibroblasts (American Type Culture Collection, CRL-12203) were transfected with RCAS-PDGF-B and RCAS-Cre plasmids, and then orthotopically injected into *Ntv-a*;*Ink4a-Arf*^−/−^;*Pten*^fl/fl^;*LSL-Luc* mice (2 months old, half male and half female, provided by E. Holland, Fred Hutchinson Cancer Research Center). Tumors were freshly isolated and subjected to mechanical dissociation with a gentleMACS Dissociator (Miltenyi) and enzymatic digestion with collagenase/hyaluronidase (Stem Cell Technologies, 07912). For lentiviral transduction, medium supernatant was collected from transfected human embryonic kidney (HEK) 293T cells expressing mouse Egfrviii, and then filtered through a 0.45-μm sterilized filter to incubate with tumor-derived sphere cells. *Cdh5-Cre*^ERT2^;*Twist1*^fl/fl^ or control *Twist1*^fl/fl^ mice (2 months old, half male and half female) were stereotactically injected with RCAS GBM tumor cells (3 × 10^5^ cells per mouse) into the brains. For the syngeneic GL261 model, mouse GL261 glioma cells (PerkinElmer, 134246) were transduced with lentiviruses encoding mouse Egfrviii and GFP, followed by flow cytometry–based cell sorting of GFP^+^ cells. GL261 glioma cells (1 × 10^5^ cells per mouse) were orthotopically injected into the brains of WT C57BL/6 mice (2 months old, half male and half female). Tumor-bearing mice were intraperitoneally treated with dimethyl sulfoxide vehicle, harmine (30 mg/kg daily for 5 consecutive days), or nanoparticles containing control or Twist1 siRNA (1 mg/kg, 2 times per week). For CAR cell therapy, mice were infused with control or Egfrviii CAR T cells (3 × 10^6^ cells per mouse) through the tail vein. Tumor growth was monitored by whole-body bioluminescence using an IVIS 200 Spectrum Imaging System after retro-orbital injection of luciferin (150 mg/kg, GoldBio). The mice were monitored for 50 days after injection and were euthanized when showing severe GBM symptoms such as domed head, hemiparesis, or a loss of more than 20% of body weight.

### Mouse CAR T cell preparation

T cells were isolated from spleens of C57/B6 mice by mechanical dissociation using the gentleMACS Dissociator (Miltenyi Biotech) and cultured in RPMI 1640 medium containing 10% FBS. Cells were treated with CD3/CD28 antibodies (5 μg/ml; BioLegend, 100302, 102102) and recombinant IL-2 (100 IU/ml; Corning, 354043) for 2 days before retrovirus transduction. Retrovirus for T cell transduction was produced by transfecting mouse Egfrviii-CAR or control CAR sequences into Phoenix cells with pMSVG and pCL-Eco helper plasmids using Lipofectamine 2000 Transfection Reagent (Life Technologies, 11668-019), followed by incubation with T cells in RetroNectin-coated plate for 2 days. For CAR expression detection, cells were immunostained with goat anti-human F(ab′)2-biotinylated antibody (Jackson Immuno-Research, 109-065-006) after transduction and analyzed by using a FACSCanto II flow cytometer (BD Biosciences).

### Nanoparticle formulation and treatment

Polymer-LNPs were generated as previously described (*21*, *22*). Briefly, C15 alkyl epoxides were reacted with PEI600 at 90°C in 100% ethanol for 48 to 72 hours at a 14:1 molar ratio. The resulting compound was purified via flash chromatography on a silica column, and then dissolved in 100% ethanol with a polyethylene glycol 2000 (PEG2000) lipid conjugate at a molar ratio of 80:20 7C1 to PEG-lipid. In vivo siRNA targeting mouse Twist1 (Thermo Fisher Scientific, 69856) or a control sequence (Thermo Fisher Scientific, 4404020) was dissolved in citrate buffer (pH 3), and then mixed in a microfluidic device with the previously described ethanol phase at a 2.5:1 flow rate ratio to form polymer-LNPs (*80*). Nanoparticles were formulated at a 5:1 weight ratio of 7C1 to siRNA. Mice were subjected to tumor induction and administrated intraperitoneally with nanoparticles (1 mg/kg) twice per week.

### Single-cell RNA-seq analysis

*Cdh5-Cre*^ERT2^;*Rosa-LSL-tdTomato* mice bearing RCAS-induced GBM tumors were euthanized and perfused with phosphate-buffered saline supplemented with EDTA. GBM tumors from three mice were harvested and digested with collagenase II (5 mg/ml; Invitrogen, 17101-015) and deoxyribonuclease (1 mg/ml, Sigma-Aldrich, D4527). Single-cell suspension was prepared after filtering using a mesh strainer with 100-μm pores. Cell samples were prepared and analyzed following a manufacturer’s V3 library protocol (10x Genomics) and scRNA-seq at the next-generation sequencing core at University of Pennsylvania. The reads were aligned with Cell Ranger (10x Genomics, version 6.1.2), and low-quality gene expression matrices were filtered out. All samples were filtered for quality control, and each cell has a number of features above 200 and below 3000 and has a percentage of mitochondrial genes below 5%. A mouse reference library (mm10) was used with tdTomato and Cre cDNA sequences to identify tdTomato^+^ and Cdh-Cre^+^ ECs. R package Seurat (version 4.0.6) was used for data analysis and visualization. For meta-analysis of human GBM scRNA-seq data, five datasets (GSE84465, GSE103224, GSE131928, GSE117891, and GSE162631) were collected from the NCBI GEO database, and cluster analysis was performed using R package Seurat (version 4.0.6).

### Bulk RNA-seq analysis

Human GBM ECs were transfected with lentivirus encoding control or Twist1 CRISPR/sgRNA. Cells were lysed in TRIzol (Thermo Fisher Scientific). RNA extraction was performed according to the manufacturer’s instructions, followed by RNA purification using an RNeasy Plus Mini Kit (Qiagen) and library construction with a TruSeq mRNA Stranded Kit (Illumina). The library was subjected to next-generation sequencing analysis with a HiSeq2500 at the Children’s Hospital of Philadelphia (CHOP). The fastq sequences were aligned to the GRCm38 reference genome using an RNA-Star aligner (v2.4.2a; https://github.com/alexdobin/STAR). The gene expression was normalized and calculated as FPKM (fragments per kilobase million) values by Cufflinks (v2.2.1) (http://cole-trapnell-lab.github.io/cufflinks/releases/v2.2.1/) with GENCODE M5 gene annotations (www.gencodegenes.org/mouse/release_M5.html).

### Mass CyTOF

Single-cell suspensions derived from freshly isolated tumors were prepared by mechanical dissociation with a gentleMACS Dissociator (Miltenyi Biotech) and enzymatic digestion with 1× collagenase/hyaluronidase (Stem Cell Technologies, 07912). After incubation with 25 μM cisplatin, cells were stained at room temperature for 30 min with heavy metal–conjugated antibodies (provided by CyTOF core at the Penn Institute for Immunology). Cells were fixed with 1.6% paraformaldehyde, stained with Cell-ID Intercalator-Ir (Fluidigm), and analyzed by a CyTOF mass cytometer (Fluidigm), followed by analysis with Cytobank (version 7.3.0), FlowJo software (V10), or R software (version 4.0.5).

### CFSE assay

Human T cells were obtained from healthy human volunteer donors by Human Immunology Core at University of Pennsylvania. The collection protocol was approved by the University of Pennsylvania Institutional Review Board, and written informed consent was obtained from each participant. T cells were incubated with CellTrace carboxyfluorescein succinimidyl ester (CFSE) solution (Thermo Fisher Scientific, C34554) for 20 min at 37°C. After treatment, cells were analyzed using a FACSCanto II flow cytometer (BD Biosciences).

### Flow cytometry

Single-cell suspensions derived from mouse tumors or human Mφs were stained with fluorescence dye-conjugated antibodies against CD11b (1:100; BioLegend 101206 or 101211), CD206 (1:100; BioLegend, 321110, 141707, or BD Biosciences, 551135), IL-10 (1:50; BD, 566568), F4/80 (1:100; Miltenyi Biotec, 130-102-327), IFN-γ (1:50; BioLegend, 502505), IL-10 (1:50; BioLegend, 505009), arginase 1 (1:50; eBioscience, 17-3697-80), or control immunoglobulin G (IgG). Cells were analyzed by using Accuri C6 (BD Biosciences) and FACSCanto II flow cytometers (BD Biosciences). The data were analyzed by FlowJo software (V10).

### Chromatin immunoprecipitation

ChIP assays were conducted using a Magna ChIP kit (Millipore, MAGNA0001) as previously described (*11*, *38*), were cross-linked with 1% formaldehyde for 10 min at room temperature, and then incubated with glycine for 5 min. Nucleic lysis was sonicated in four cycles (each for 8 × 2 s; interval, 45 s) using a W-385 sonicator (Heat Systems Ultrasonics). Immunoprecipitation was performed by using anti-Twist1 (20 μg; Abcam, ab50887), anti-SATB1 (20 μg; Cell Signaling Technology, 8067), or control IgG (20 μg; Millipore, 12-371 or PP64) with protein A–conjugated beads. Immunoprecipitants along with inputs acquired from 1% sheared DNA were reverse–cross-linked and purified. The primer pairs used are listed as follows. For *SATB1* ChIP: #1 (FP: 5′-AACACGCGCACTCCTCCTCT-3′, RP: 5′-CCCTAGTGGGGAAGCATACA-3′), #2 (FP: 5′-CTTGTTGTTTGGCTGGGTTT-3′, RP: 5′-GTCTCGACCGAACATTGACGG-3′), #3 (FP: 5′-AGACGATGTTTCCTAGAGGG-3′, RP: 5′-ACTAATGGGCTGGGTCACAG-3′), #4 (FP: 5′-CTGTGACCCAGCCCATTAGT-3′, RP: 5′-CCCTTCTCTCCCAATGTCAA-3′), #5 (FP: 5′-GAACCTTCCCAAGTGGAGAC-3′, RP: 5′-TTTCCCAAAGGCTACACAAT-3′), #6 (FP: 5′-CCTCTTCTTTGTACGCTTGT-3′, RP: 5′-TGGGGACTTAATGAGAGAGCA-3′), #7 (FP: 5′-AGAGAGGAGGAAACTATGGAA-3′, RP: 5′-CTTGAAAGTCGAATCAACCA-3′), #8 (FP: 5′-CTCTGCTGTATTTTTGTGTGC-3′, RP: 5′-GACCTGTGGGGACAGAAAGT-3′), #9 (FP: 5′-GTGTCTGAGAAAGACATCAGG-3′, RP: 5′-GTATTCTAACAGAGCCCATG-3′), #10 (FP: 5′-TTATGCAATTCTGGGTGCAG-3′, RP: 5′-CCAGATTAAATGCCCCTTTC-3′). For *SPP1* ChIP: #1 (FP: 5′-CAGACTTCCCTCCACTAAAT-3′, RP: 5′-TAGGGGGAAATATGTCTCCC-3′), #2 (FP: 5′-GGCAAAAGGAAGCTGACACTT-3′, RP: 5′-GATAGCAGAGCTCTGGGTCC-3′), #3 (FP: 5′-CTAATATTCGGACTTTCCCTG-3′, RP: 5′-GAGAGTAGACGTAAGATCTT-3′), #4 (FP: 5′-GGACATTACAATTCGTGACTGC-3′, RP: 5′-GCCTACCTATCCTTGTTCCCT-3′), #5 (FP: 5′-GCCCAAGGTTGCACAGGTCA-3′, RP: 5′-CGAGTATGCAGTAGCTTGTTAC-3′), #6 (FP: 5′-CTTGAGTAGTAAAGGACAGAGG-3′, RP: 5′-GAGCACTTAGGGATCCCATG-3′), and #7 (FP: 5′-CCTGGATGCTGAATGCCCAT-3′, RP: 5′-ACACAGGGAGGCGGAGAGATT-3′).

### Immunoblot

Cells were lysed with an NP-40 lysis buffer containing a protease inhibitor cocktail (Roche, 11697498001). Cell lysates were centrifuged at 12,000*g* for 15 min at 4°C, and supernatants were collected. Supernatants were diluted with Laemmli SDS sample buffer, and protein samples (20 μg) were resolved by 10 to 15% SDS–polyacrylamide gel electrophoresis (Bio-Rad). Proteins were transferred into polyvinylidene difluoride membranes and blocked with TBST buffer containing 5% dried milk. The membranes were blotted with anti-Twist1 (1:1000; Santa Cruz, sc-81417), anti-OPN (1:1000; R&D Systems, MAB14331-SP), anti-Snail (1:1000; Cell Signaling Technology, 3879), anti-Satb1 (1:100, Cell Signaling Technology, 8067), or anti-GAPDH (1:3000, Cell Signaling Technology, 5174) antibody overnight at 4°C. Proteins were detected with goat anti-rabbit or anti-mouse IgG-HRP conjugate (1:5000; Bio-Rad, 1706515 or 1706516) and developed with ECL (GE Healthcare, RPN2232).

### siRNA transfection

Human GBM-derived ECs were transfected with siRNAs targeting Twist1 (Thermo Fisher Scientific, s14523), Snail (Thermo Fisher Scientific, s13185), Satb1 (Thermo Fisher Scientific, 106912), or control siRNA (Thermo Fisher Scientific, 4390843) using Lipofectamine 2000 Transfection Reagent (Life Technologies, 11668-019) in serum-free Opti-MEM medium (Gibco, 31985-070) for 12 hours, followed by incubation with serum-supplemented medium for 48 hours. For in vivo treatment, cells were incubated with LNPs containing Twist1 siRNA (Thermo Fisher Scientific, 69856) or control siRNA (Thermo Fisher Scientific, 4404020).

### CRISPR/sgRNA transduction

The guide RNA sequence targeting Twist1 or random sequences was subcloned into lentiCRISPR v2 puro vector (Addgene, 52961). The DNA oligos for subcloning sgRNA sequences were listed as follows: #1 (FP: 5′-CACCGCTACGCCTTCTCGGTCTGG-3′, RP: 5′ AAACCCAGACCGAGAAGGCGTAGC-3′), #2 (FP: 5′-CACCGCTGTCGTCGGCCGGCGAGAC-3′, RP: 5′-AAACGTCTCGCCGGCCGACGACAGC-3′), #3 (FP: 5′-CACCGCGGGAGTCCGCAGTCTTACG-3′, RP: 5′-AAACCGTAAGACTGCGGACTCCCGC-3′), and #4 (FP: 5′-CACCGATCTCTCGAGCGGCGACGCG-3′, RP: 5′-AAACCGCGTCGCCGCTCGAGAGATC-3′). HEK293T cells (Sigma-Aldrich, 12022001) were transfected with mixed lentiCRISPR and packing vectors. After centrifuging to remove the cell debris, the supernatant containing the virus was filtered through membranes with 0.45-μm pores. Human GBM ECs were transduced with the lentivirus expression Twist1 sgRNA with polybrene (8 μg/ml; Millipore) for 24 hours.

### CUT&RUN assay

Human GBM patient–derived ECs were analyzed by CUT&RUN assay using a CUTANA kit (Version 3, EpiCypher, 14-1048), following the manufacturer’s instruction. Briefly, 5 × 10^5^ cells per reaction were collected, washed, and mixed with activated ConA beads, followed by incubation with 0.5 μg of IgG or anti-Twist1 antibody (20 μg; Abcam, ab50887) at 4°C overnight. K-MetStat Panel was spiked before the addition of the antibody. Cells were incubated with PAG-micrococcal nuclease, and the reaction was terminated by stop buffer with *Escherichia coli* Spike-in DNA. Solubilized chromatin fragments were released and purified. Isolated DNA was enriched for library preparation and sequenced by Illumina NextSeq (Center for Applied Genomics, CHOP). CUT&RUN data were processed using the nf-core/cutandrun pipeline. Specifically, reads were trimmed to 50 bases with barcodes removed, followed by alignment using Bowtie2. Duplicates were removed using Picard. Peak calling was performed by MACS2 and visualized by R package ChipSeeker and Gviz.

### Statistical analysis

Statistical analysis was performed using Prism software (GraphPad, version 9.0). Two-sided Student’s *t* test or one-way analysis of variance (ANOVA) for comparisons with two or more than two groups, respectively. Kaplan-Meier survival curves were generated, and a log-rank test was used to evaluate the statistical significance between groups. A *P* value lower than 0.05 was considered significant.
