# Supplementary material for: An immunosuppressive vascular niche drives macrophage polarization and immunotherapy resistance in glioblastoma
Source: Sci Adv. 2024 Feb 28;10(9):eadj4678. doi: 10.1126/sciadv.adj4678 (PMC10901371; doi:10.1126/sciadv.adj4678)
Supplement: Supplementary file 1 — Figs. S1 to S7 [file sciadv.adj4678_sm.pdf]

Science Advances
AAAS

Supplementary Materials for

An immunosuppressive vascular niche drives macrophage polarization and immunotherapy resistance in glioblastoma

Fan Yang *et al.*

Corresponding author: Yi Fan, fanyi@upenn.edu; Yanqing Gong, gongy@pennmedicine.upenn.edu

Sci. Adv. **10**, eadj4678 (2024)
DOI: 10.1126/sciadv.adj4678

This PDF file includes:

Figs. S1 to S7

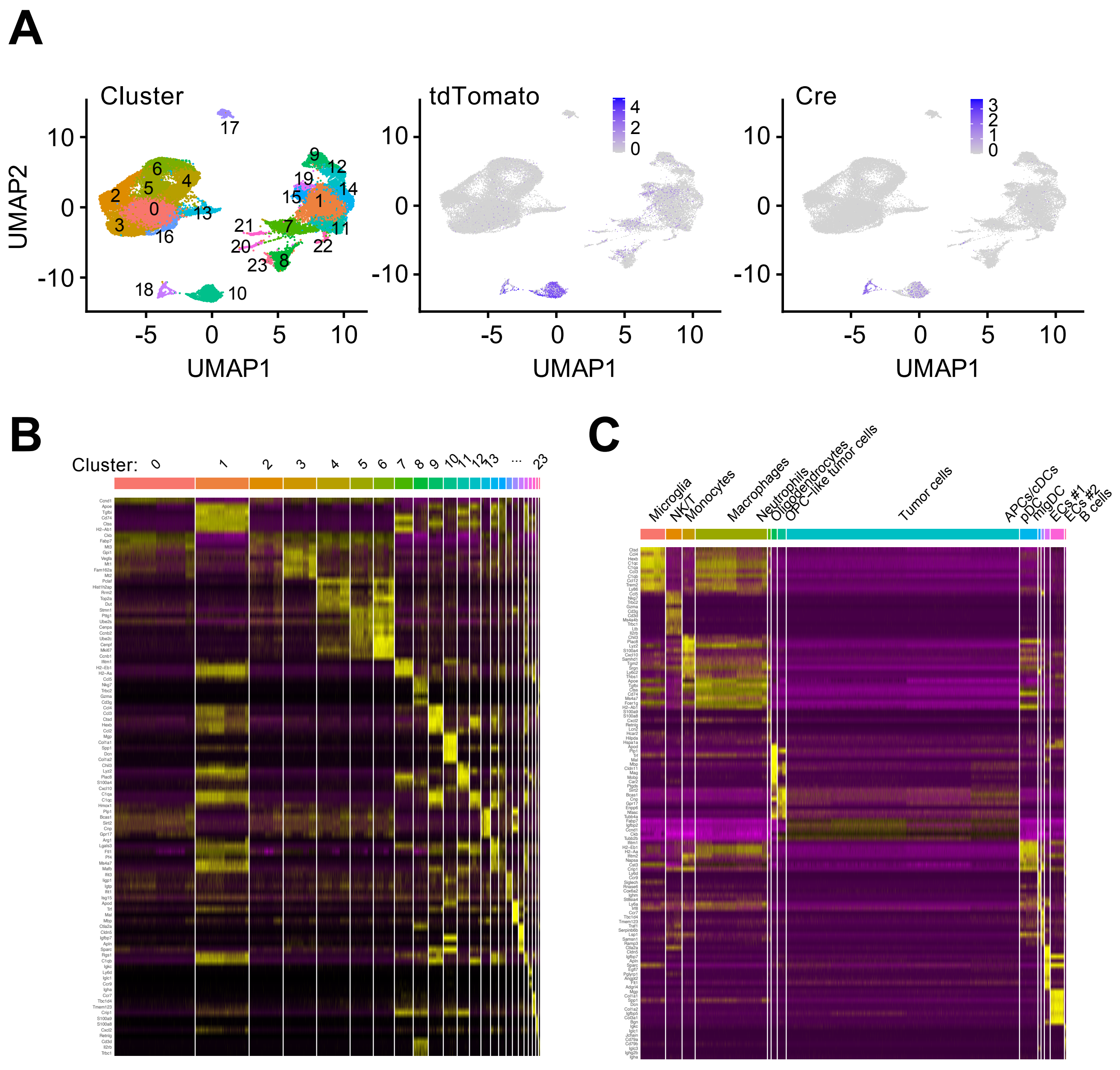

Fig. S1. single-cell RNAseq analysis of mouse GBM tumors with endothelial lineage tracing.
GBM was induced in *Rosa-LSL-tdTomato*;*Cdh5-Cre*^{ERT2} mice (n = 3 mice). Tumors were excised and analyzed by single-cell RNAseq. (**A**) UMAP analysis of transcriptome gene signature assigned cells into different clusters with tdTomato and Cre expression. Expression heatmaps of top upregulated genes for (**B**) all clusters and (**C**) identified cell types are shown.

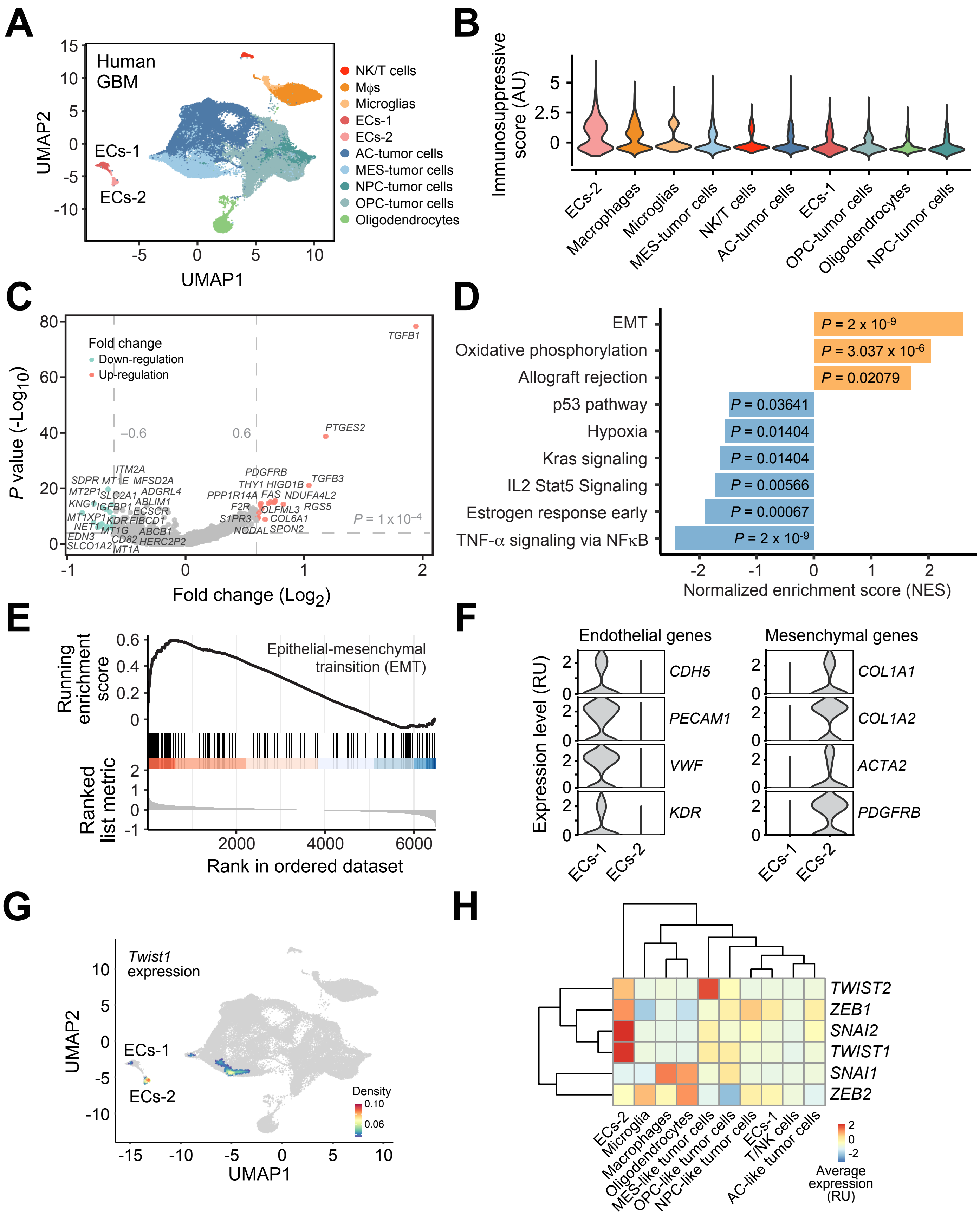

Fig. S2. Identification of a mesenchymal-like population of ECs with Twist1 expression in human GBM. Meta-analysis of human single-cell RNA-seq data sets (n = 27 human GBM tumors). (**A**) UMAP analysis of transcriptome gene signature assigned cells into different clusters. (**B**) Immunosuppressive score in different cell clusters was analyzed based on the average expression of immunosuppressive molecules. (**C**-**E**) Genes with altered expression were identified in tumor ECs with high immunosuppressive scores, compared with ECs with low immunosuppressive scores. (**C**) Top upregulated and downregulated genes. (**D**) Normalized enrichment scores (NES) were calculated for top enriched pathway analysis. (**E**) Enrichment analysis of EMT pathway. (**F**) Expression distribution of endothelial- and mesenchymal-associated genes in two EC populations. (**G**,**H**) Expression distribution of EMT-associated transcriptional factors was analyzed in all cell clusters. (**G**) UMAP analysis of Twist1 expression. (**H**) Expression profiles of Snail, Slug, Twist1/2, and Zeb1/2.

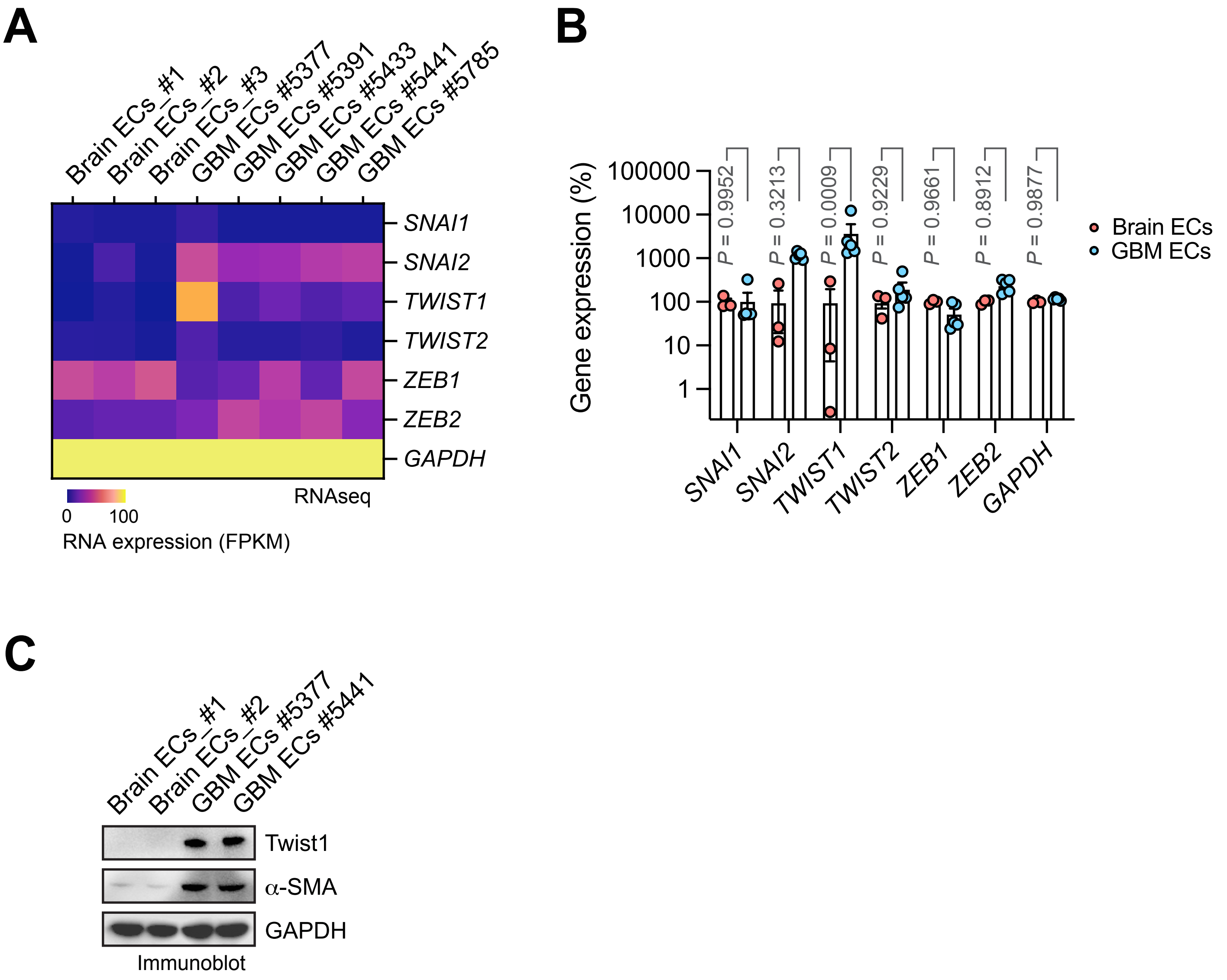

Fig. S3. Expression of EMT-associated transcriptional factors in normal brain- and tumor-derived ECs.
Human ECs were isolated from normal brains or GBM tumors. (**A**,**B**) Cells were subjected to bulk RNA-seq analysis (Zhang et al, *Cell Metabolism*, 2023;35(3):517-534, PMID: 36804058). Expression of EMT-associated transcriptional factors was analyzed (n = 3-5 humans). (**A**) heatmap of gene expression. (**B**) Quantified results. Statistical analysis by two-way ANOVA. (**C**) Cell lysates were analyzed by immunoblot analysis.

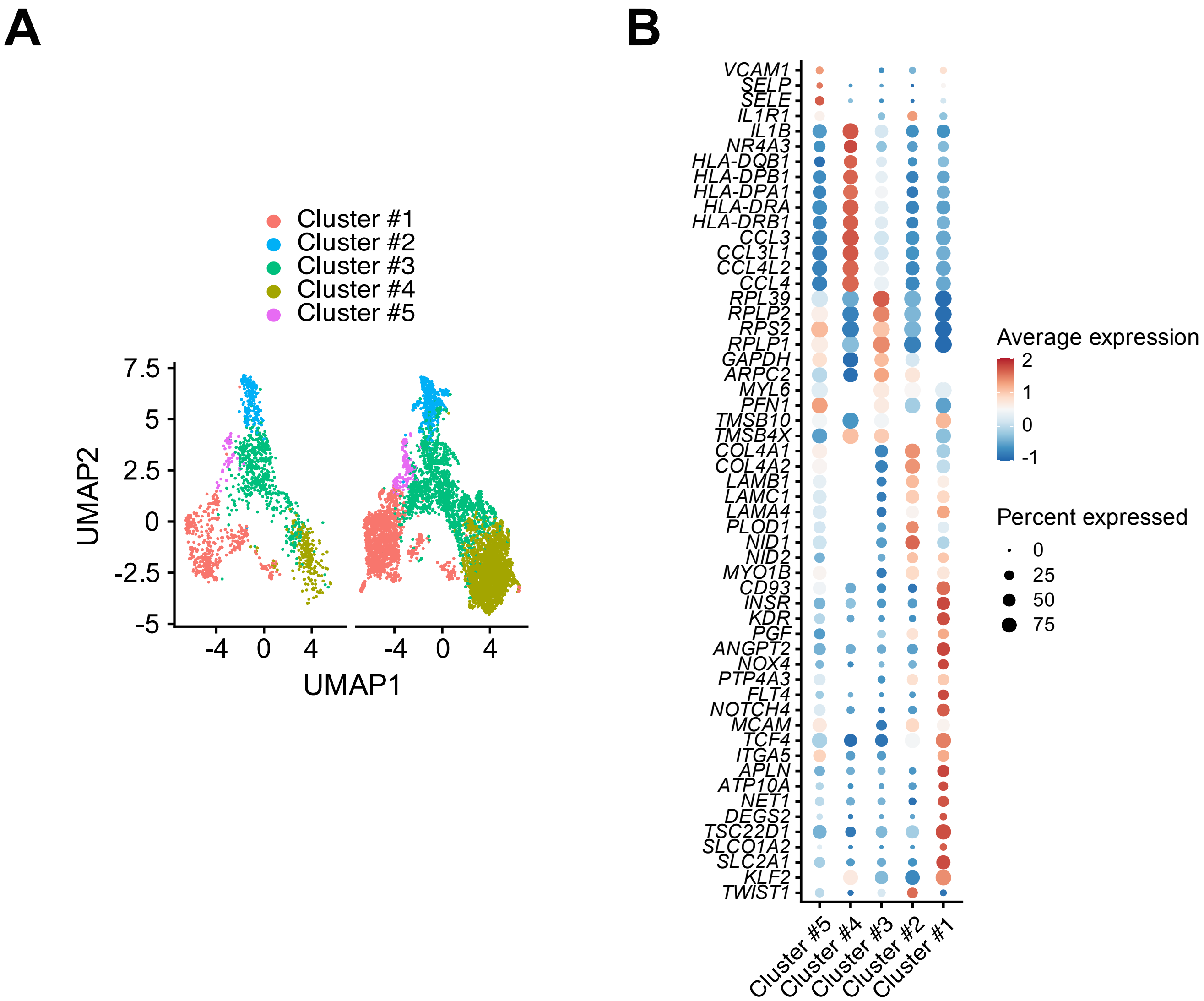

Fig. S4. Twist1 expression in a subpopulation of tumor ECs.
Single-cell RNAseq data of mouse tdTomato⁺ tumor ECs were integrated with published single-cell RNAseq data of human tumor ECs. (**A**) UMAP analysis of transcriptome gene signature assigned cells into 5 different clusters. Left, our data. Right, the published data (Xie et al, *JCI Insight*, 2021;6(15):e150861. PMID: 34228647). (**B**) Expression of published signature genes and Twist1 in different cell clusters.

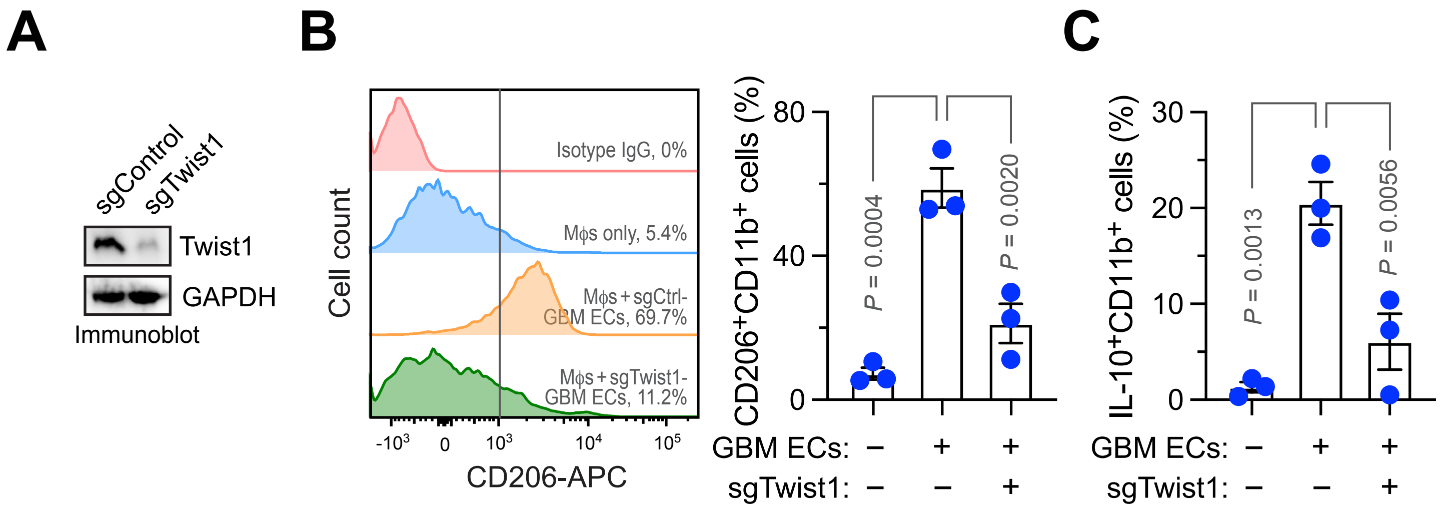

Fig. S5. CRISPR/sgRNA-mediated Twist1 knockdown inhibits EC-induced alternative Mϕ polarization. GBM ECs isolated from human patients were transduced with lentivirus encoding control or Twist1 CRISPR/sgRNA. (**A**) Cell lysate was immunoblotted. (**B**,**C**) Treated ECs were incubated with human PBMC-derived Mϕs. CD206 and IL-10 expression in CD11b$^+$ Mϕs was analyzed by flow cytometry. (**B**) CD206 expression. Left, representative sortings. Right, quantified results (n = 3 human Mϕ samples, mean ± SEM). Statistical analysis by one-way ANOVA. (**C**) Quantified results of IL-10 expression (n = 3 human Mϕ samples, mean ± SEM). Statistical analysis by one-way ANOVA.

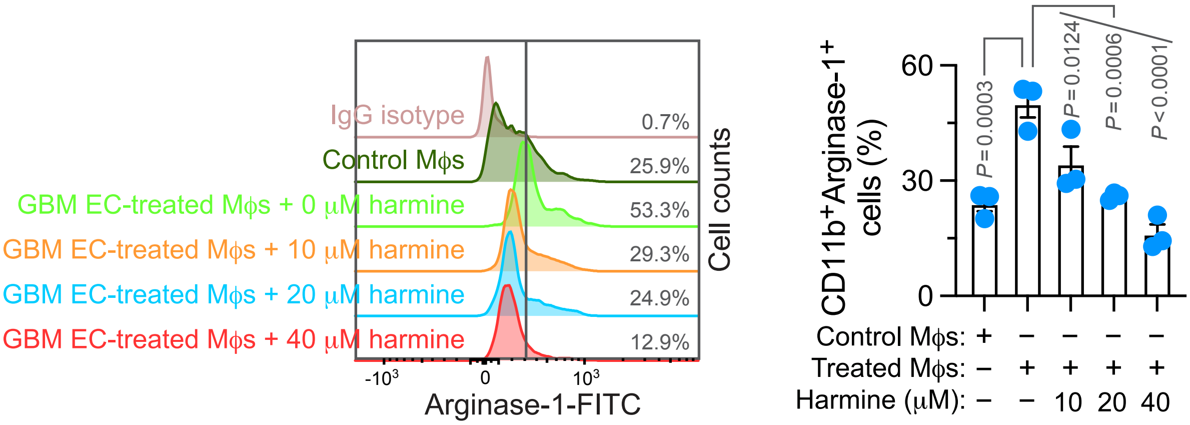

Fig. S6. Twist1 inhibition reduces arginase-1 expression in Mϕs.

Human PBMC-derived Mϕs were incubated with or without GBM ECs in the presence or absence of harmine. Arginase-1 expression in CD11b⁺ Mϕs was analyzed by flow cytometry. Left, representative sortings. Right, quantified results (n = 3 human samples, mean ± SEM). Statistical analysis by one-way ANOVA.

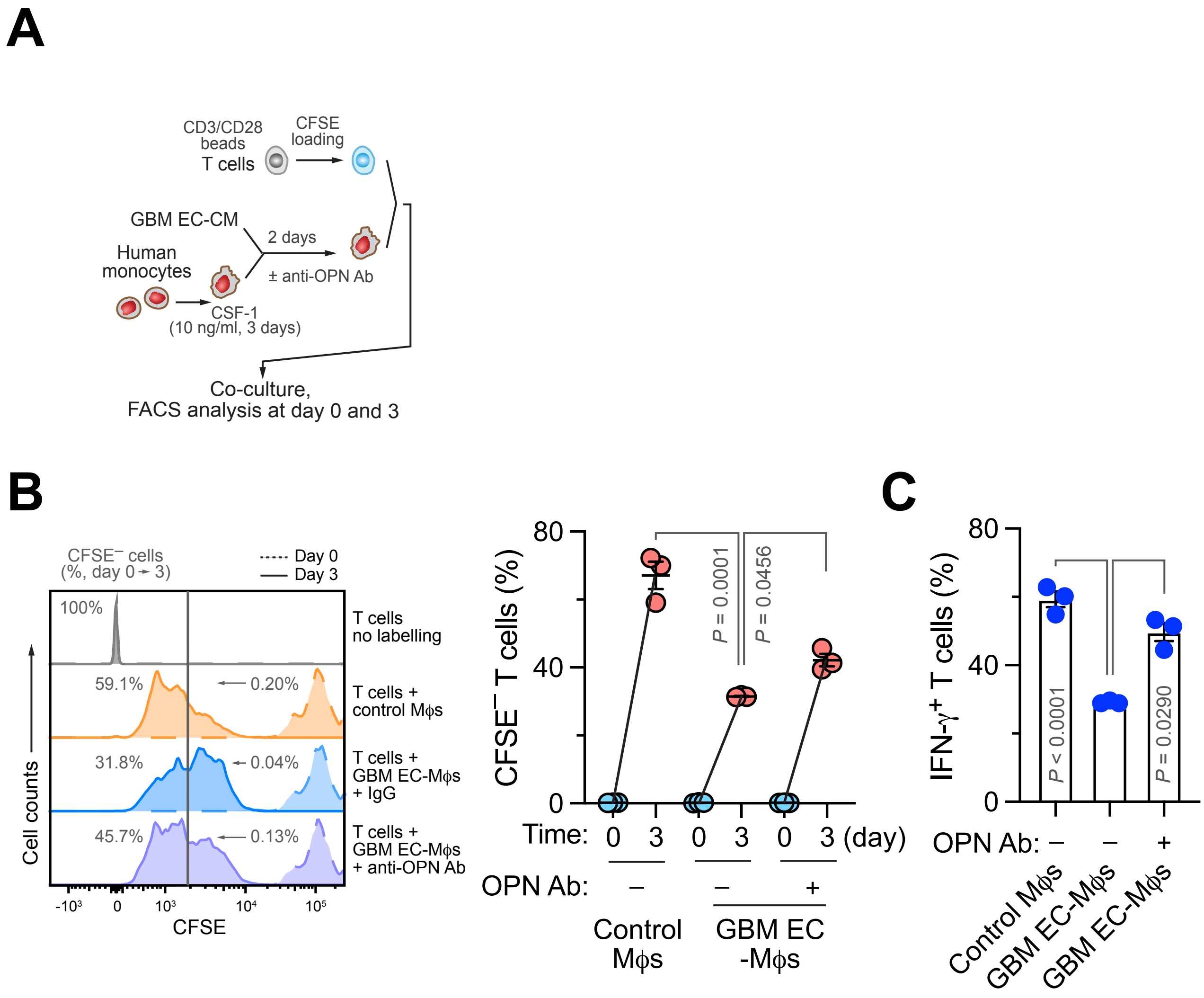

Fig S7. OPN is critical for T cell inactivation induced by GBM EC-educated Mϕs.
CFSE-loaded human T cells were incubated with human PBMC-derived T cells that were pretreated with or without GBM EC-conditioned medium. (**A**) Experimental procedure. (**B**) CFSE was analyzed in $CD3^+$ T cells at day 0 and day 3. Left, representative cell sortings. Right, quantified results (n = 3 human samples, mean ± SEM). Statistical analysis by one-way ANOVA. (**C**) IFN-γ was analyzed in $CD3^+$ T cells. Shown are quantified results (n = 3 human samples, mean ± SEM). Statistical analysis by one-way ANOVA.
